# Biomimetic nanoparticles targeting atherosclerosis for diagnosis and therapy

**DOI:** 10.1002/SMMD.20230015

**Published:** 2023-08-03

**Authors:** Yuyu Li, Jifang Wang, Jun Xie

**Affiliations:** ^1^ Department of Cardiology National Cardiovascular Disease Regional Center for Anhui the First Affiliated Hospital of Anhui Medical University Hefei China; ^2^ Key Laboratory of Remodeling‐Related Cardiovascular Diseases, Ministry of Education, Beijing Collaborative Innovation Centre for Cardiovascular Disorders, Beijing Anzhen Hospital, Capital Medical University Beijing China; ^3^ Beijing Institute of Heart, Lung, and Blood Vessel Diseases Beijing Anzhen Hospital Affiliated to Capital Medical University Beijing China; ^4^ Department of Cardiology Drum Tower Hospital Medical School of Nanjing University Nanjing China

**Keywords:** atherosclerosis, nano‐diagnosis, nanoparticles, nano‐therapy

## Abstract

Atherosclerosis is a typical chronic inflammatory vascular disease that seriously endangers human health. At present, oral lipid‐lowering or anti‐inflammatory drugs are clinically used to inhibit the development of atherosclerosis. However, traditional oral drug treatments have problems such as low utilization, slow response, and serious side effects. Traditional nanodrug delivery systems are difficult to interactively recognize by normal biological organisms, and it is difficult to target the delivery of drugs to target lesions. Therefore, building a biomimetic nanodrug delivery system with targeted drug delivery based on the pathological characteristics of atherosclerosis is the key to achieving efficient and safe treatment of atherosclerosis. In this review, various nanodrug delivery systems that can target atherosclerosis are summarized and discussed. In addition, the future prospects and challenges of its clinical translation are also discussed.

## Graphical abstract

The design principles and applications of biomimetic nanoparticles for diagnostics and therapy of atherosclerosis. With proper engineering, various types of cells (lymphocytes, monocytes, neutrophils, platelets, macrophages, stem cells, etc.) have been used as a source of coating materials to prepare cell membrane‐based nanotherapies. Applicable nanoparticle cores include organic nanoparticles (polymers), inorganic nanoparticles (silica nanoparticles, metal nanoparticles), hybrid nanoparticles (MOFs), etc. By taking advantage of the inherent property of source cells, these biomimetic nanotherapeutics confer a wide range of functions in diagnostics and therapy for atherosclerosis.

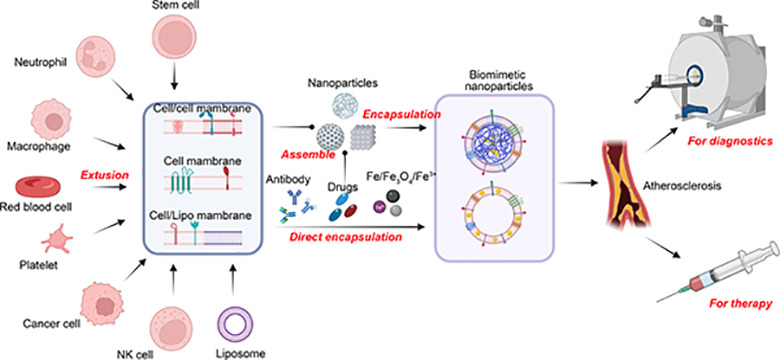

**Key points**

- Nano‐diagnosis and nano‐therapy for atherosclerosis are becoming increasingly advanced and diversifying.
- Development of nanoparticle drug delivery systems can effectively carry drugs to atherosclerotic plaques for achieving targeted therapies.
- Nanomaterials and nanotechnology have great potential value in clinical translation and applications.

## 1 INTRODUCTION

Atherosclerosis is the most common underlying pathological factor for coronary artery disease, peripheral artery disease and cerebrovascular disease,^1^, ^2^ which refers to the accumulation of fat or fibrous material in the arterial intima. Over time, this chronic accumulation of vascular plaques in the subendothelial layer of the large and middle arteries makes them increasingly fibrotic, accumulates calcium minerals and produces calcification.^3^, ^4^ Advanced atherosclerotic plaques eventually lead to severe stenosis, restrict blood flow and cause severe tissue hypoxia, causing harm to human health.^5^, ^6^ Atherosclerotic cardiovascular disease (CVD) includes myocardial infarction, strokes, and peripheral artery disease. Remains the leading cause of vascular disease worldwide. At present, the treatment of atherosclerosis is very limited. Although statin lipid‐lowering drugs have achieved certain effects, new therapeutic drugs are still in urgent need of invention. Therefore, a number of biomimetic nanoparticles have been developed to target atherosclerotic plaques for diagnosis and treatment.

The classic hypothesis of dyslipidemia is already well known.^7^, ^8^ However, the occurrence and development of atherosclerosis is not only caused by dyslipidemia. With the deepening of research, the immune system has been discovered to play a crucial role in atherosclerosis.^4^, ^9^ In atherosclerosis, a variety of factors (including immune, pathogenic, hemodynamic, etc.) stimulate endothelial cell damage and activation.^10^, ^11^, ^12^ Activated inflammatory endothelial cells highly express a series of adhesion molecules and chemokines, such as vascular cell adhesion molecule‐1 (VCAM‐1), intercellular adhesion molecule‐1 (ICAM)‐1, E‐selectin and P‐selectin, thereby causing the recruitment of monocytes in the blood and leading monocytes to invade and accumulate in the blood vessel wall.^13^, ^14^ Monocytes ingest the deposited oxidized lipids in the arterial wall and then differentiate into macrophages and cause a series of inflammatory reactions, which in turn lead to the further development of atherosclerosis.^15^, ^16^ In addition, a large number of studies have also established the role of adaptive immunity in the formation of atherosclerosis.^17^, ^18^, ^19^ Human atherosclerotic lesions contain activated T lymphocytes, which indicate the activation of adaptive immunity.^19^, ^20^ Some T‐cell subtypes, such as type 1 T helper (Th1) cells, accelerate the development of atherosclerosis, while other subtypes, such as regulatory T (Treg) cells, appear to alleviate the progression of atherosclerosis.^21^, ^22^ Moreover, many basic experiments, mainly in mice, have strictly demonstrated the causal role of adaptive immunity in regulating experimental atherosclerosis.^19^, ^23^ These findings, as well as the study of human atherosclerotic plaques, provide direct evidence for the development of inflammation and the immune response in atherosclerosis.

Based on the continuous understanding of the mechanism of atherosclerosis, a large number of innovative nanobiomaterials have also been continuously developed for the diagnosis and treatment of atherosclerosis. Especially in the research and development of biomimetic nanoparticles, various ligands were designed to modify the surface to give it various biological functions.^24^, ^25^ The designed biomimetic drug delivery systems exhibited many excellent characteristics, such as solubilizing drugs, increasing the drug half‐life period, improving drug distribution in the body, enhancing targeting, reducing toxicity and side effects, etc.^26^, ^27^ It has great application potential in the field of diagnosing and treating atherosclerosis.

## 2 NANOMATERIALS

**FIGURE 1:**
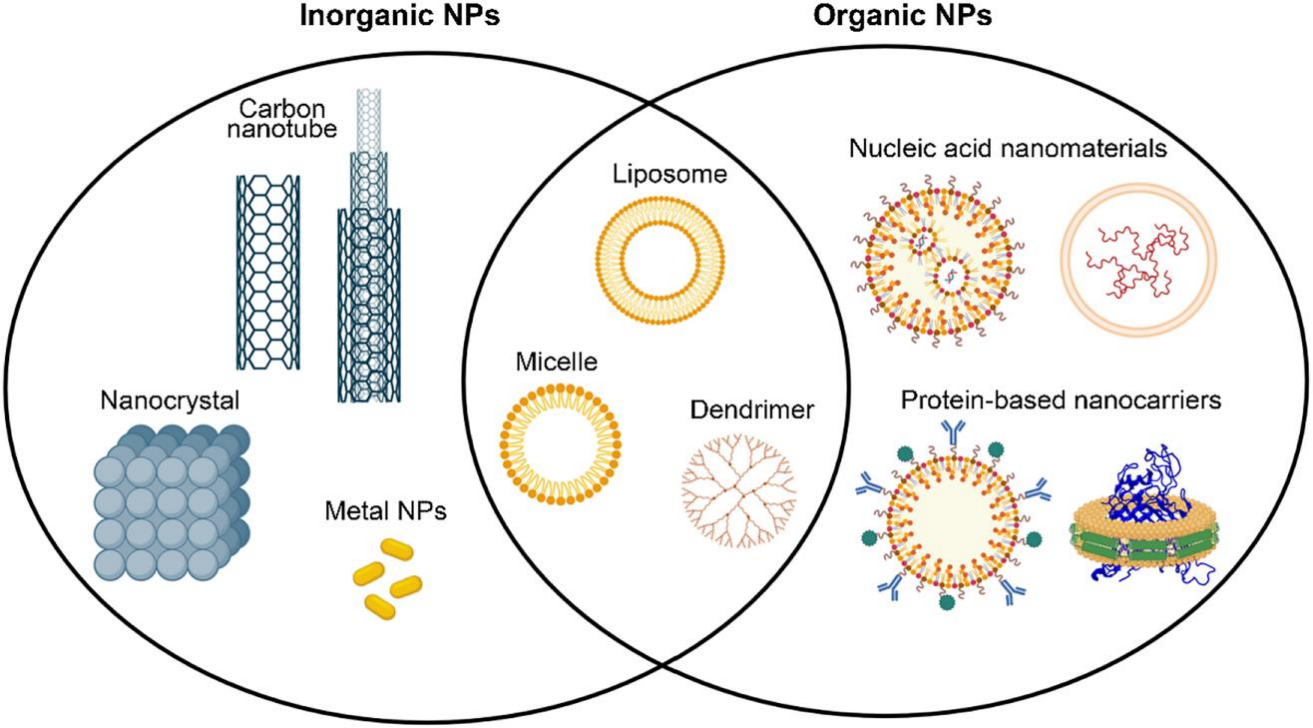
Schematic illustration of rough classification and structure of partial nanomaterials.

Nanomaterials were defined as “natural, incidental or manufactured material containing particles, in an unbound state or as an aggregate or as an agglomerate and where for 50% or more of the particles in the number size distribution, one or more external dimensions are in the size range 1–100 nm.”^28^ According to the shape and morphology of nanomaterials, they can be divided into three dimensions: 0‐dimensional (nanoparticles),^29^ 1‐dimensional (nanowires) nanomaterials,^30^ and 2‐dimensional (nanolayers),^31^ which are smaller than 100 nm in all directions, one axis, or two axes, respectively.^32^ Nanomaterials have unique physical (minuscule size, high surface energy, magnetic effects, large specific surface area, etc.), chemical (high reactivity, catalytic ability, resistance to corrosion, etc.) and biological properties (biocompatibility, low immunogenicity, biodegradability, etc.).^33^ According to their unique properties and dimensions, nanomaterials can imitate natural nanoscale extracellular matrix components and directly deliver biologically active substances. Nanoparticles (NPs) can pass through cell membranes and help cells absorb protein. Based on the above considerations, nanomaterials with natural properties or functionalized with other suitable features can be constructed and applied in tissue engineering,^34^ drug delivery,^35^ bioimaging,^36^ gene therapy,^37^ and other fields (Figure 1).

### 2.1 Inorganic nanomaterials

Inorganic nanoparticles include metals, metal oxides, carbon nanomaterials, etc.^38^ Their potential for biomedical applications includes but is not limited to drug and gene delivery, antimicrobial agents, labeling or imaging in cells and tissues, and applications in diagnostic and therapeutic diagnostics.^39^, ^40^

#### 2.1.1 Carbon nanotubes

Carbon nanotubes (1 nm in diameter and 1–100 nm in length) are carbon‐based well‐ordered cylindrical molecules that consist of a single layer of carbon atoms (graphene sheet) rolled into a cylinder. The configuration of carbon nanotubes includes multiwalled or single‐walled configurations or nanotubes interlinked concentrically that are composed of C60 fullerenes.^41^ Due to their high surface area, mechanical strength, excellent thermal and electrical conductivity, and superior thermal and chemical stability, carbon nanotubes exhibit superiority as nonpolymeric drug carriers, imaging contrast agents, and biosensors.^42^, ^43^ Carbon nanotubes with hollow tube‐like structures hold molecules via capillary action and adsorption, and functionalization allows the carbon nanotubes to conjugate a variety of biomolecules, such as carbohydrates, proteins, peptides, and therapeutic and diagnostic devices. Carbon nanotubes provide economical, powerful and accurate diagnostic and therapeutic value.^44^

Mahsa et al. combined ultraselective nanoparticle targeting of Ly‐6C^hi^ monocytes and foamy macrophages with clinically viable photoacoustic imaging (PAI).^45^ PAI identified that inflamed atherosclerotic plaques displayed an ∼6‐fold greater signal than controls (*p* < 0.001) 6 h after intravenous injection of ultraselective carbon nanotubes, with in vivo corroboration via optical imaging. Carbon nanotubes are useful for the accurate identification and diagnosis of atherosclerotic lesions. Alyssa et al. developed a macrophage‐specific nanotherapy based on single‐walled carbon nanotubes loaded with a chemical inhibitor of the antiphagocytic Cluster of differentiation 47(CD47)‐Signal regulatory protein alpha (SIRPα) signaling axis.^46^ They demonstrated that these single‐walled carbon nanotubes accumulated within the atherosclerotic plaques, reactivated lesional phagocytosis and reduced the plaque burden in atheroprone apolipoprotein‐E‐deficient mice without compromising safety, thereby overcoming a key translational barrier for this class of drugs.

#### 2.1.2 Metal nanoparticles (metal NPs)

Metal NPs have unique antibacterial properties, catalytic activity, and immunoregulatory functions. Metal NPs of precious metals have been widely used in fields of medicine, resulting from their abovementioned natural features.

Magnetic NPs not only have the characteristics of nanomaterials (such as a large specific surface area, high coupling capacity and small size) but also have magnetic responsiveness and superparamagnetic properties, as they gather and locate in a magnetic field, absorb electromagnetic waves and produce heat in an alternating magnetic field.^47^ Therefore, magnetic nanomaterials could be used to deliver drugs to specific targets.

Based on the characteristics of magnetic nanoparticles, studies on their imaging in atherosclerosis have received extensive attention. Magnetic nanoparticles can target atherosclerotic plaques because they can be taken in rapidly by macrophages. In predominantly rupture and rupture‐prone human atherosclerotic lesions, accumulation of ultrasmall superparamagnetic iron oxide (USPIOs) in macrophages results in reduced signal in magnetic resonance imaging (MRI) in vivo.^48^ In addition, some magnetic nanoparticles that are adorned with certain biomolecules can target plaques by binding to biomolecules within the atherosclerotic plaque, such as ligand‐conjugated iron oxide nanoparticles (IONPs) for MRI of endothelial adhesion molecules (VCAM‐1 and P‐selectin).^49^, ^50^

### 2.2 Organic nanomaterials

Organic nanomaterials mainly include polymer nanoparticles and nanomaterials based on natural biological macromolecules, such as nucleic acid nanomaterials, protein nanomaterials, and polypeptide nanomaterials.

#### 2.2.1 Nucleic acid nanomaterials

Nucleic acids (DNA and RNA) are fundamental “molecules of life,” as they play essential roles in gene heredity, regulation, and expression. More importantly, both of these molecules have remarkable sequence recognition capabilities and can be conveniently synthesized with a nearly infinite number of sequences. All these unique properties have made DNA and RNA intriguing building blocks for nanoengineering.

##### Construction of nucleic acid nanoparticles

Linear DNA lacks complexity and flexibility, and the algorithmic assembly strategy proposed by Rothemund et al. catalyzes the production of more sophisticated and stable nucleic acid structures.^51^ In addition, DNA origami nonperiodic two‐dimensional architectures, such as rectangles and squares, presented by Rothermond in 2006,^52^ can build various large structures instead of tiles as foundation units.^53^ Many applications in nanomedicine and nanorobotics require additional capabilities, such as controlled three‐dimensional assembly and movement. Currently, scientists have attempted to engineer three‐dimensional nucleic acid objects. The engineering design of three‐dimensional nanostructures of nucleic acids includes the strategy of origami assembly,^54^ piling up multilayer origami structures,^55^ etc.

Unfortunately, constructing DNA assemblies that are also functional remains difficult. Relying on the synergistic self‐assembly of RNA and DNA, incorporating RNA is a promising strategy to circumvent this issue^56^ as RNA is structurally related to DNA but exhibits rich chemical, structural and functional diversity; mRNAs carry information that directs protein syntheses, and rRNAs fold and assemble into ribosomes. Ribozymes can catalyze chemical reactions, aptamers can specifically bind to ligands, and microRNAs and interfering RNA (siRNA) regulate gene activities. This strategy may enable the assembly of both RNA and DNA into large nanostructures with multifunctional RNA modalities.

##### Applications of nucleic acid nanostructures in atherosclerosis

**FIGURE 2:**
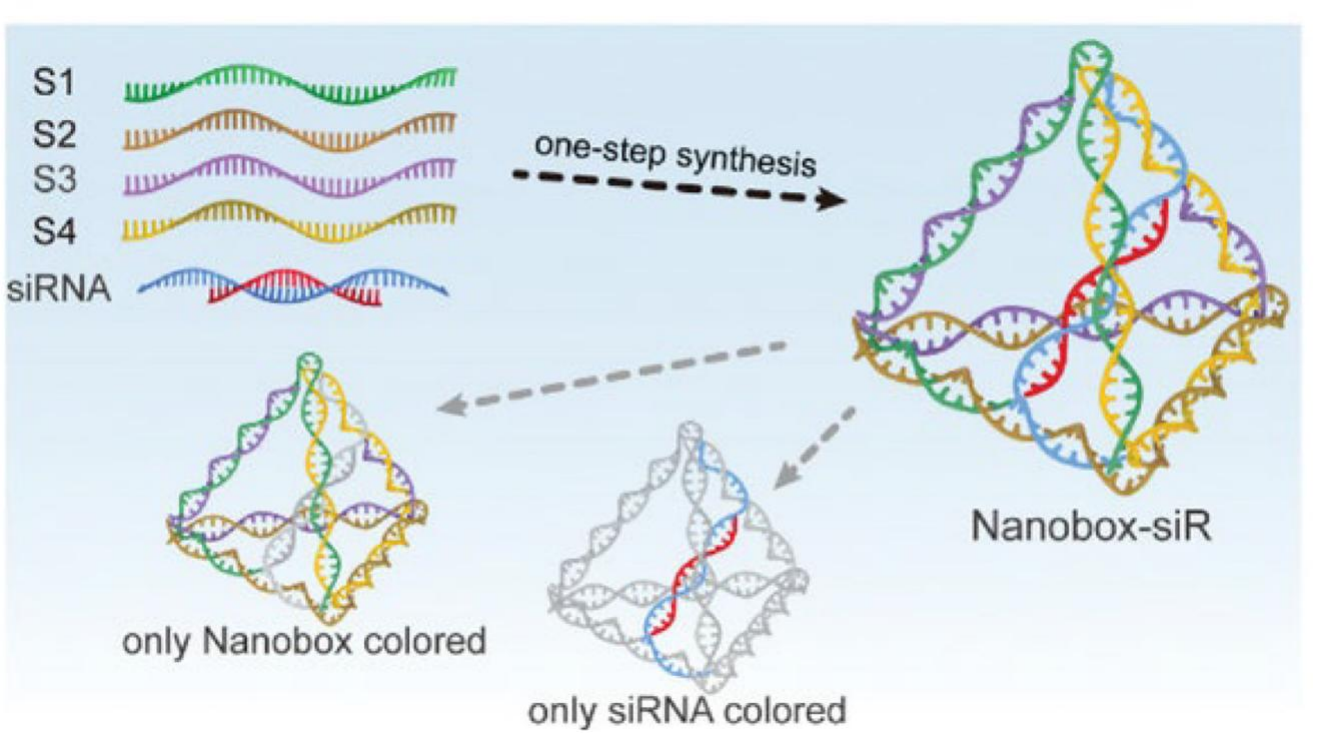
Schematic showing nanobox‐siR synthesis. Reproduced with permission.^57^ Copyright 2022, John Wiley and Sons.

Regarding the applications of DNA, a few researchers have applied some kinds of tetrahedral DNA nanomaterials to encapsulate and dynamically control target. Gao et al. fabricated a dynamic lysosome‐activated tetrahedral DNA nanobox, completely encapsulating siRNA with greater resistance to RNase and serum and enabling solid integration with the vehicle during delivery (Figure 2).^57^ They upgraded a tetrahedral nucleic acid nanobox that was matched with an extended antisense strand of siRNA to completely embed the cargo siRNA and further perform encapsulation and protection tasks. However, it is unfortunate that the instability of RNA has made many scientists flinch away from RNA nanotechnology, though RNA molecules not only can be designed and manipulated with a level of simplicity characteristic of DNA but also possess versatility in structure and function similar to that of proteins. Only a few reports have explored rationally designed RNA nanoparticles based on the three‐way junction (3WJ) in the field of targeted cancer diagnosis and therapy, demonstrating promising future applications due to their thermal stability and molecular‐level plasticity.^58^

Although its full potential remains to be explored, nucleic acid nanoengineering is nothing short of revolutionary. The implications of being able to precisely manipulate the position and orientation of materials at the atomic level are enormous. It could potentially lead to a plethora of novel materials and products that can impact nanoelectronics, aerospace and nanomedicine.^53^

#### 2.2.2 Protein‐based nanocarriers

In nature, there are abundant protein resources, including various antibodies and virus capsids, which are both natural proteins and natural nanomaterials. Nanomaterial research is essential to proteins because of their exceptional characteristics, namely biodegradability, nonantigenicity, high nutritional value, abundant renewable sources and extraordinary binding capacity of various drugs. Protein‐based nanostructure studies focus on the binding of proteins to polymer chains to increase their size and block the attachment of transporters to the recognition areas on the surface of nanocarriers for efficient uptake by cells. Protein‐based nanocarriers represent promising candidates for efficient drug and gene delivery.

##### Applications of protein‐based nanomaterials

The detection of early‐stage atherosclerotic plaques has been the goal of many scholars. Vascular cell adhesion molecule‐targeted protein nanoparticles give us hope. In vivo experiments with murine atherosclerosis models indicated that the targeted protein nanoparticles that target VCAM‐1 were successful in detecting plaques of various sizes in the descending aorta and the aortic arch.^59^ Additionally, targeting VCAM‐1, the (99 m)Tc‐labeled, anti‐VCAM1 nanobody cAbVCAM1‐5 could be a new class of radiotracers for cardiovascular applications.^60^

Generally regarded as safe (GRAS) drug delivery devices have high nutritional value and are rich in renewable resources. Regarding safety, they are metabolizable in vivo by digestive enzymes into innocuous peptides. Additionally, protein‐based nanoparticles exhibit high loading capacity of various drugs due to multiple binding sites present in their molecules. They exhibit a variety of possible drug loading mechanisms, including electrostatic attractions, hydrophobic interactions, and covalent bonding. Moreover, protein‐based nanoparticles offer various possibilities for surface modification due to the presence of functional groups (i.e., carboxylic and amino groups) on the surface of the nanoparticles, thus enabling a specific drug targeting to the site of action.^61^ For example, native nanostructured lipoproteins such as low‐ and high‐density lipoproteins (LDL and HDL) are powerful tools for the targeted delivery of drugs. Yonghong Luo et al. generated a phospholipid‐based and high‐density lipoprotein like nanoparticle, miNano. They found that miNano accumulated in atherosclerotic plaques and colocalized with cholesterol crystals (CCs) and macrophages in vivo. Administration of miNano inhibited atherosclerosis and improved plaque stability.^62^ Lin Di also proposed that due to the importance and high prevalence of oxidized LDL (oxLDL)‐dependent mechanisms of uptake in atherosclerosis, oxLDL mimetics or oxLDL‐like carriers were particularly interesting for their use in atherosclerosis.^63^

Our research on the application of protein‐based nanomaterials in atherosclerosis is limited, and their advantages in atherosclerosis have broad application prospects.

### 2.3 Polymeric nanoparticles

Polymeric nanoparticles are capable of loading antibodies, DNA or RNA, allowing particular interactions in individual targets. The degradation products have no toxicity, and absorbable parts metabolize with no need for surgical removal intervention once the agent delivery is depleted.

On the basis of polymeric nanoparticles, researchers have further synthesized amphiphilic block copolymer nanoparticles. Amphiphilic block copolymers are formed by covalent binding of hydrophobic and hydrophilic blocks possessing single or multiple groups of hydrophilic/hydrophobic units. Various types of nanoparticles are formed by varying the ratio of the hydrophobic/hydrophilic blocks. These amphiphilic copolymers offer several advantages, such as optimal drug solubilization, particle size, and stability during administration and transport. Furthermore, amphiphilic block copolymers are also amenable to functionalization.

#### 2.3.1 Component substances of polymeric nanoparticles

Polyethylene glycol (PEG) and poly (lactic‐glycolide acid) (PLGA) are commonly used in the synthesis of polymer nanoparticles. PEG is a typical polymeric nanoparticle. Polyethylene glycolized nanoparticles mean that polyethylene glycol is added to the surface of the nanoparticles to provide a hydration layer and space barrier around the polymer.^64^ It reduces nonspecific binding of serum proteins, prolongs circulation and retention time, reduces proteolysis and renal excretion,^65^ protects antigenic determinants from immune detection, and thus reduces cellular clearance by the mononuclear phagocyte system. The combination of lactic acid (LA) with glycolic acid (GA) results in a copolymer system known as PLGA. Its polymeric properties, such as its glass transition temperature (Tg) and degradation rate, can also be fine‐tuned by modifying the LA:GA content ratio.^66^ PLGA can be used to load various types of drugs, such as hydrophilic or hydrophobic, small or large molecules.^67^

#### 2.3.2 Applications of polymeric nanoparticles in atherosclerosis

Magnetic resonance contrast agents encapsulated by polymeric nanoparticles and then cleverly surface‐modified can accumulate better in atherosclerotic lesions and assist in the diagnosis of atherosclerosis. Marie‐Josée et al. proposed an original targeted contrast agent for molecular imaging of atherosclerosis. Versatile ultrasmall superparamagnetic iron oxide (VUSPIO) nanoparticles enhancing contrast in MRI were functionalized with a recombinant human IgG4 antibody, rIgG4 TEG4 by targeting human activated platelets.^68^ High‐resolution ex vivo MRI demonstrated the selective binding of TEG4‐VUSPIO to atheroma plaques. In addition, polymeric nanoparticles can make drugs safer and more precise to use. In the study by Emanuela et al., an advanced pharmaceutical formulation for Rapa administration in which Rapa was encapsulated in a polymer with a surface modified with phosphatidylcholine that targeted macrophages was designed and optimized to be extemporaneously dispersed in a physiological medium, giving rise to a colloidal dispersion of nanoparticles ready to be administered in vivo.^69^

## 3 CELL MEMBRANE‐COATED BIOMIMETIC NANOPARTICLES

**FIGURE 3:**
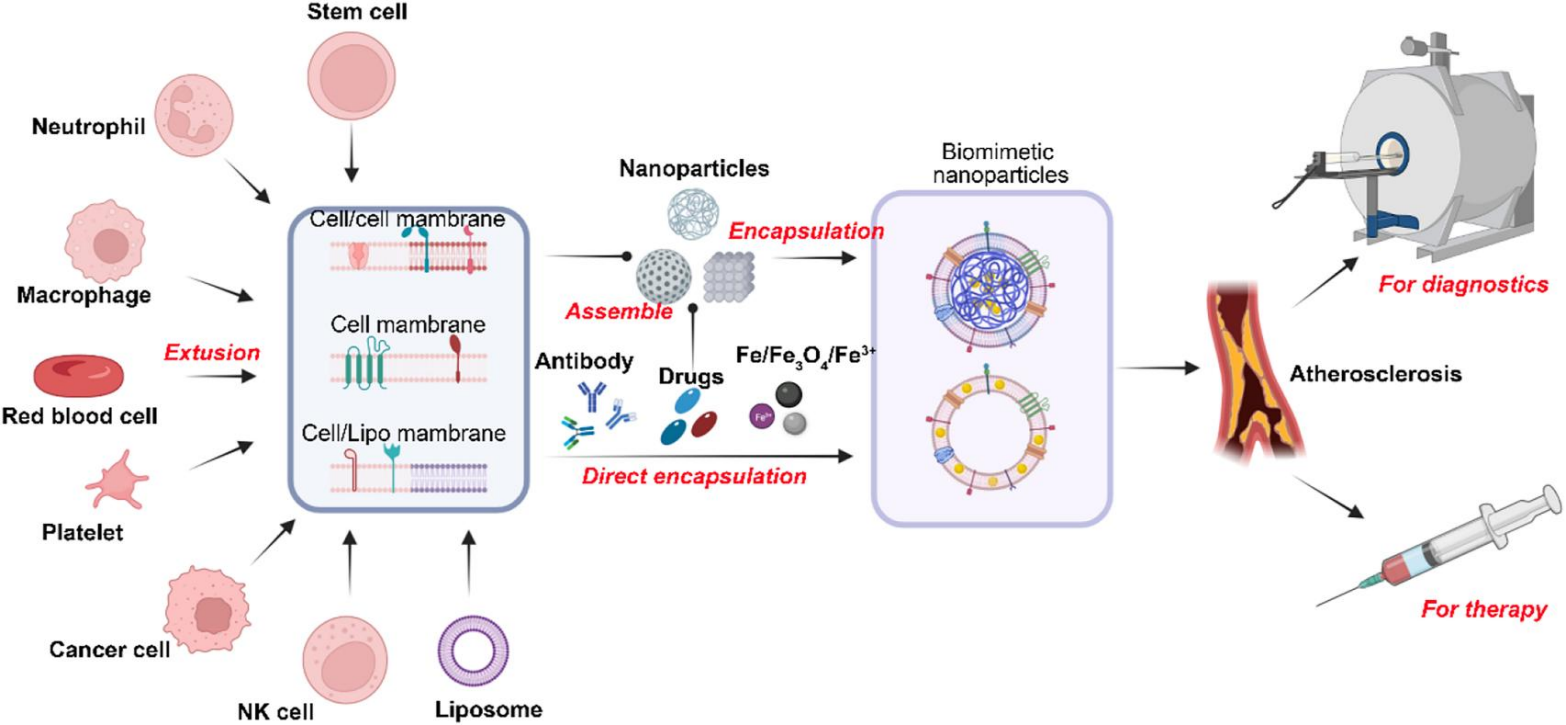
Schematic illustration of the design principles and applications of biomimetic nanoparticles for diagnostics and therapy of atherosclerosis.

Biomimetic nanoplatforms are a recent and emerging strategy that plays an important role in a wide variety of applications. The different types of membranes used for coating include membranes from red blood cells, platelets, leukocytes, neutrophils, cancer cells, macrophages, etc. Membrane‐coated nanoparticles indeed mimic source cells and play an important role in the diagnosis and treatment of many diseases (Figure 3).

### 3.1 Macrophages

Macrophages are phagocytes derived from monocytes, and their morphology changes with different functional stages.^70^ Macrophages are mainly involved in the human body's innate immunity and eliminate invading pathogens.^71^ In addition, in the adaptive immune response, macrophages can be used as antigen‐presenting cells, which can present antigens to T cells to activate the adaptive immune response.^72^ The promotion and development of atherosclerosis is closely related to inflammation, in which macrophages can respond to chemotactic signals and migrate to the site of inflammation.^73^, ^74^ This chronic inflammation homing ability gives macrophages great potential as drug delivery vehicles.

#### 3.1.1 Mechanism of targeting to atherosclerotic plaque based on macrophage membranes

In atherosclerosis, the vascular endothelial cells in the plaque are in an activated state and highly express the vascular adhesion molecule VCAM‐1. These adhesion molecules can specifically bind to integrins (integrin α4/β1) on the membranes of circulating monocytes. Through the VCAM‐1/integrin system, circulating monocytes are recruited into atherosclerotic plaques. Based on this principle, macrophage membrane‐functionalized biomimetic nanoparticles have been designed to attach to atherosclerotic plaques.

#### 3.1.2 Diagnosis of atherosclerosis based on macrophage membranes

**FIGURE 4:**
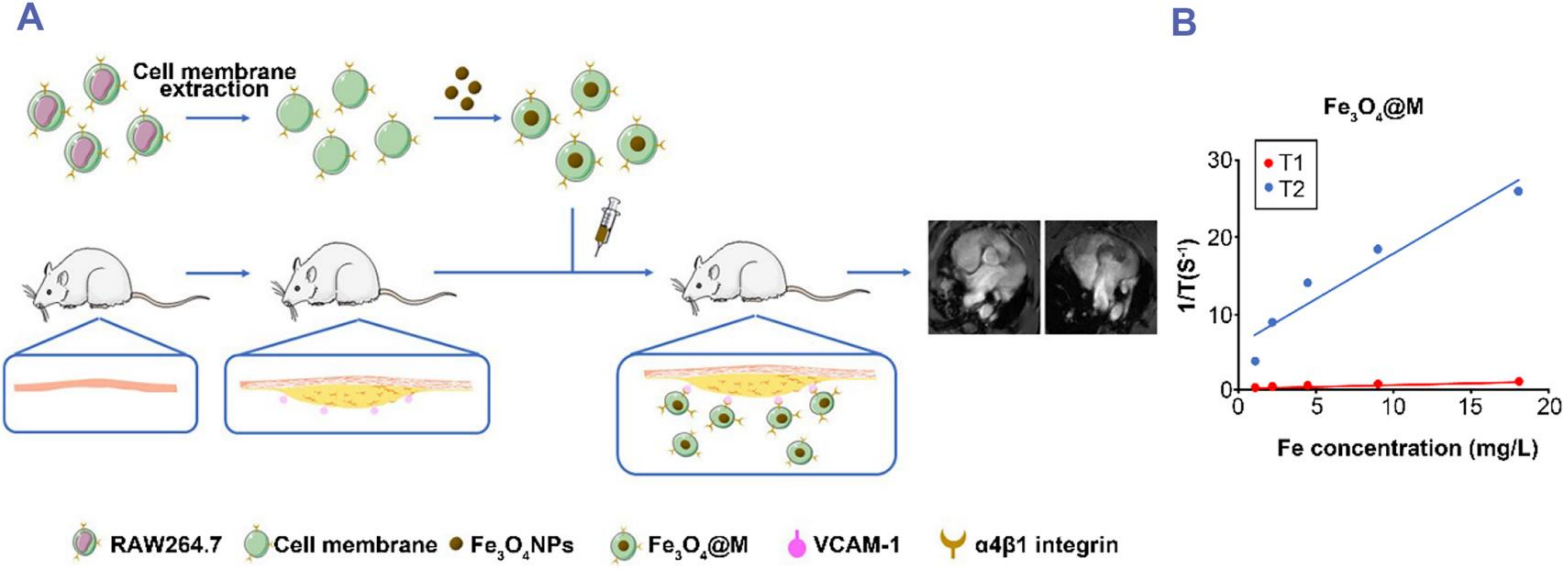
Macrophage membrane‐based drug delivery system for atherosclerotic diagnosis. (A) Schematic illustration of the preparation of macrophage membrane‐coated Fe_3_O_4_ nanoparticles (Fe_3_O_4_@M) for detecting early lesions of atherosclerosis (foam cells). (B) T1 and T2 relaxation times of PEG‐coated Fe_3_O_4_ nanoparticles (Fe_3_O_4_@PEG) and Fe_3_O_4_@M. Reproduced with permission.^75^ Copyright 2021, Elsevier.

In a study of biomimetic nanoparticles for the diagnosis of atherosclerosis, Xin Huang et al. designed Fe_3_O_4_ biomimetic nanoparticles coated with a macrophage membrane (Fe_3_O_4_@M), which could investigate the imaging effect on the early lesions of atherosclerosis. The results show that Fe_3_O_4_@M nanoparticles have high biological safety and can target the early lesions of atherosclerosis. This was found to be safe and effective in vivo MRI contrast agent (Figure 4).^75^

#### 3.1.3 Treatment of atherosclerosis based on macrophage membranes

**FIGURE 5:**
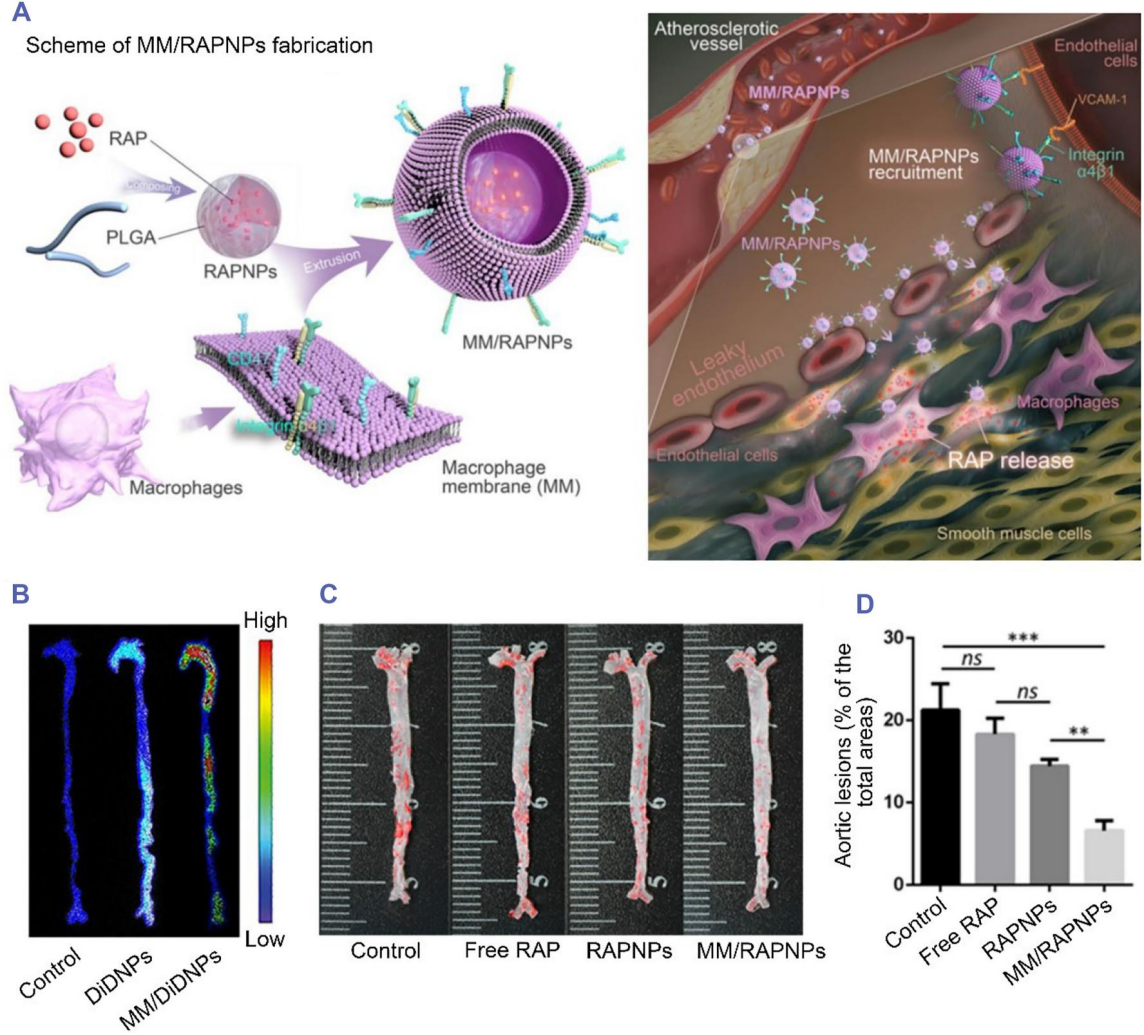
Macrophage membrane‐based drug delivery system for atherosclerotic therapy. (A) Schematic of macrophage membrane‐coated rapamycin nanoparticle (MM/RAPNP) fabrication and its treatment for atherosclerosis. (B) Atherosclerotic mice were injected with DiD‐labeled nanoparticles (DiDNPs) and macrophage‐coated DiD‐labeled nanoparticles (MM/DiDNPs). Representative ex vivo fluorescence images of DiD fluorescent signals accumulated in the aorta 24 h postinjection. (C) Representative photographs of en face ORO‐stained aortas with different treatments (treated with free rapamycin [Free RAP], rapamycin nanoparticles [RAPNPs] and macrophage membrane‐coated rapamycin nanoparticles [MM/RAPNPs]). (D) Quantitative analysis of the lesion area (*n* = 5, mean ± SD). **p < 0.01, ***p < 0.001 and ns, no significance. Reproduced under terms of the CC‐BY license.^76^ Copyright 2021, The Authors, published by Ivyspring International Publisher.

For atherosclerosis therapy, Yi Wang et al. prepared macrophage membrane‐functionalized biomimetic nanoparticles loaded with rapamycin (RAP). First, rapamycin‐loaded PLGA nanoparticles (RAPNPs) were prepared. Then, the macrophage membrane (MM) was extracted from RAW 264.7 cells to coat the RAPNPs, forming the drug delivery system MM/RAPNPs. Compared with RAPNPs or free RAP, MM/RAPNPs could effectively reduce atherosclerotic lipid deposition and inhibit the progression of atherosclerosis. Moreover, the MM/RAPNPs showed a good safety profile and long‐term drug release characteristics in mice.^76^ These results indicate that macrophage membrane‐coated biomimetic nanoparticles have potential clinical application value for atherosclerosis therapy (Figure 5).

### 3.2 Platelets

Platelets can be thought of as nonnucleated cells released by megakaryocytes. When the body undergoes an inflammatory response due to injury, platelets usually accumulate at the injury sites and interact with other blood cells to help stop bleeding and stabilize blood vessels.^77^, ^78^ Platelets have a small size, their diameter is approximately 2–4 μm, and they are easily activated by mechanical or chemical stimulation.^79^ Activated platelets change shape and produce platelet‐derived microparticles. Recent studies have shown that platelets have certain natural targeting properties to surgical wounds, circulating tumor cells and inflammation sites.^80^, ^81^ In addition, platelets have certain immune functions, can secrete chemokines, and can recruit and activate T cells and other immune cells to participate in inflammatory reactions.^82^, ^83^ All of these factors provide a theoretical basis for platelets to be used in the preparation of biomimetic nanodrug delivery systems.

#### 3.2.1 Mechanism of targeting to atherosclerotic plaque based on platelets membranes

Platelets play a vital role in the occurrence and acceleration of atherosclerosis.^84^, ^85^ Studies have shown that in the early stages of atherosclerosis, Platelet glycoprotein (GP) Ibα on the platelet membrane can be combined with Von Willebrand Factor (vWF) expressed by injured vascular endothelial cells, which allows platelets to be recruited to atherosclerotic plaques.^86^, ^87^ As atherosclerosis progresses, vascular endothelial cells are intensely damaged, the collagen under the endothelium is continuously exposed, and GP Ia‐IIa and GP VI on the platelet membrane can adhere to atherosclerotic plaques through interactions with collagen.^85^ In addition, platelets can express P‐selectin, CD40L and ICAM‐2, which can directly interact with P‐selectin glycoprotein ligand‐1(PSGL‐1), CD40 and Lymphocyte function‐associated antigen 1 (LFA‐1) on inflammatory cells.^88^, ^89^, ^90^ The presence of these adhesion molecules allows the platelet membrane to be designed for targeted therapy and diagnosis of atherosclerosis.

#### 3.2.2 Diagnosis of atherosclerosis based on platelets membranes

**FIGURE 6:**
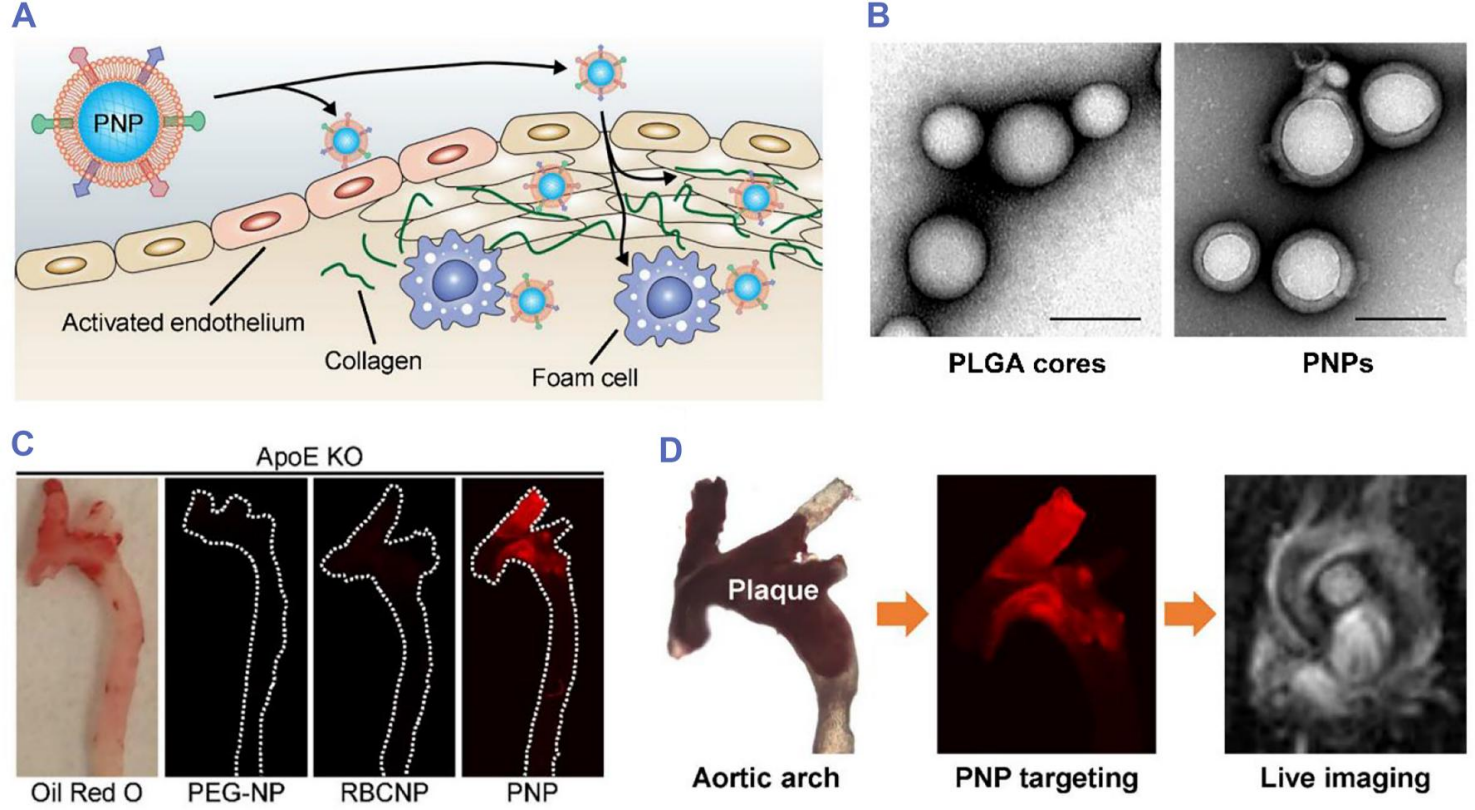
Platelet membrane‐based drug delivery system for atherosclerotic diagnosis. (A) Schematic illustration of the targeted delivery of PNPs. PNPs express a variety of surface markers capable of targeting different components of atherosclerotic plaques, including activated endothelium, foam cells, and collagen. (B) Transmission electron microscopy (TEM) image of bare PLGA cores (left) and PNPs (right) negatively stained with uranyl acetate (scale bars = 100 nm). (C) Macroscopic fluorescent imaging of aortic arches from ApoE^−/−^ mice fed a high‐fat western diet after intravenous administration of PEG‐coated nanoparticles (PEG‐NPs), red blood cell membrane‐coated nanoparticles (RBCNPs), or platelet membrane‐coated nanoparticles (PNPs) (white = physical outline, red = nanoparticle). (D) T1‐weighted MRI images of ApoE KO mice before and 1 h after administration of PNP‐loaded lipid‐chelated gadolinium (Gd‐PNPs) (orange arrows = regions of positive contrast along the aortic arch). Reproduced with permission.^91^ Copyright 2018, American Chemical Society.

Inspired by the efficient recruitment of platelets into atherosclerotic plaques. Xiaoli Wei et al. designed platelet membrane‐coated nanoparticles (PNPs). Then, lipid‐chelated gadolinium (Gd) was inserted into the lipid bilayer of the platelet membrane to form the Gd‐PNPs. The ApoE^−/−^ mice were scanned before and 1 h after administration of Gd‐PNPs by T1‐weighted MRI. The results indicated that the Gd‐PNPs could accumulate in the underlying biology of the targeted regions.^91^ Gd‐PNPs may have the potential to become a novel targeted contrast agent that could target atherosclerotic plaques in clinical diagnosis (Figure 6).

#### 3.2.3 Treatment of atherosclerosis based on platelets membranes

**FIGURE 7:**
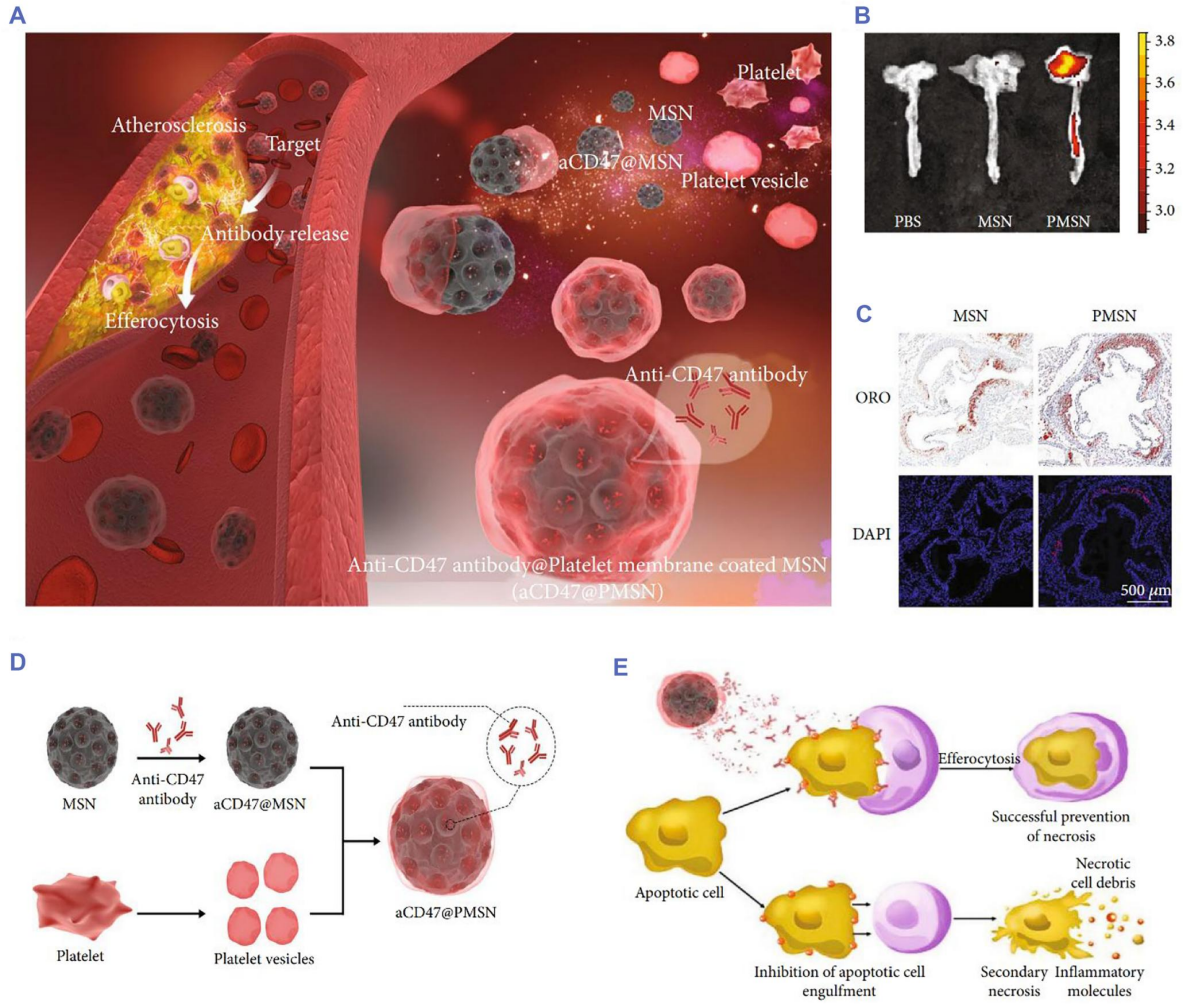
Platelet membrane‐based drug delivery system for atherosclerotic therapy. (A) Illustration of platelet membrane‐coated mesoporous silicon nanoparticles targeting plaques to deliver CD47 antibody for atherosclerotic treatment. (B) Accumulation of mesoporous silicon nanoparticles (MSN) and platelet membrane‐coated mesoporous silicon nanoparticles (PMSN) in atherosclerotic plaques of ApoE^−/−^ mice observed by Ex Vivo FL Imaging. (C) Tissue sections of atherosclerotic plaques in ApoE^−/−^ mice injected with MSN and PMSN (blue: DAPI; red: MSN‐RITC and PMSN‐RITC (D) Illustration of the platelet membrane‐coated mesoporous silicon nanoparticle fabrication process. (E) Illustration of a platelet membrane‐coated mesoporous silicon nanoparticle carrying anti‐CD47 antibody (CD47@PMSN) promoting phagocytosis of necrotic smooth muscle cells. Reproduced under terms of the CC‐BY license.^94^ Copyright 2022, The Authors, published by American Association for the Advancement of Science.

For the treatment of inflammatory disease with platelet membrane‐coated biomimetic nanoparticles, Che‐Ming J. Hu et al. successfully extracted the platelet membrane from human (blood type O) peripheral venous blood. PNP was designed to adhere to pathogens.^92^ The biomimetic nanoparticles showed good immune targeting properties and provided a novel approach for disease‐targeting delivery. Recently, Yanan Song et al. used PNP to deliver the RAP to atherosclerotic plaques. RAP‐PNP has been proven to have a better targeting ability and a more significant therapeutic effect than free RAP or RAP‐NP.^93^ Furthermore, Liang Chen et al. developed platelet membrane‐coated mesoporous silicon nanoparticles (PMSN) as a drug delivery system to target atherosclerotic plaques with the delivery of an anti‐CD47 antibody (CD47@PMSN). Compared with free anti‐CD47 antibody, CD47@PMSN significantly prevented the necrotic decay of cells but significantly promoted the efferocytosis of necrotic cells in the plaques (Figure 7).^94^

### 3.3 Red blood cells

Red blood cells are the largest component of blood cells and can survive for approximately 70–140 days under the physiological state in the body.^95^ The main function of red blood cells is to transport oxygen and carbon dioxide.^96^ In addition, red blood cells adhere to the antigen‐antibody complex through the complement receptor proteins on the cell surface and carry the complex to the liver and spleen for clearance.^97^ Since the red blood cell membrane has unique characteristics, it is also widely used in the preparation of biomimetic materials.

#### 3.3.1 Mechanism of targeting to atherosclerotic plaque based on red blood cells membranes

When vasculature supportive tissue intimates were absent, the formation of leaky vessels and pores (100 nm to 2 μm in diameter) was promoted. In addition, the lymphatic circulation was restricted leading to increased retention, causing macromolecular substances and particles to have selective high permeability and retention.^98^, ^99^ This phenomenon is an enhanced permeability and retention (EPR) effect, which has been widely studied in the field of oncology.^100^, ^101^, ^102^ Taking advantage of the EPR effects, various nanoparticles with complex functions have been studied for the treatment of cancer.^103^, ^104^, ^105^ In fact, the EPR effect also exists in atherosclerotic lesions.^106^, ^107^ The leaky endothelium and capillaries in atherosclerotic plaques allow nanoparticles to penetrate the blood vessel wall and accumulate in pathological lesions.^106^, ^107^ Among them, the red blood cell membrane has been widely used in nanomedicine research due to its excellent biocompatibility, long half‐life and wide sources.^108^, ^109^

#### 3.3.2 Treatment of atherosclerosis based on red blood cells membranes

**FIGURE 8:**
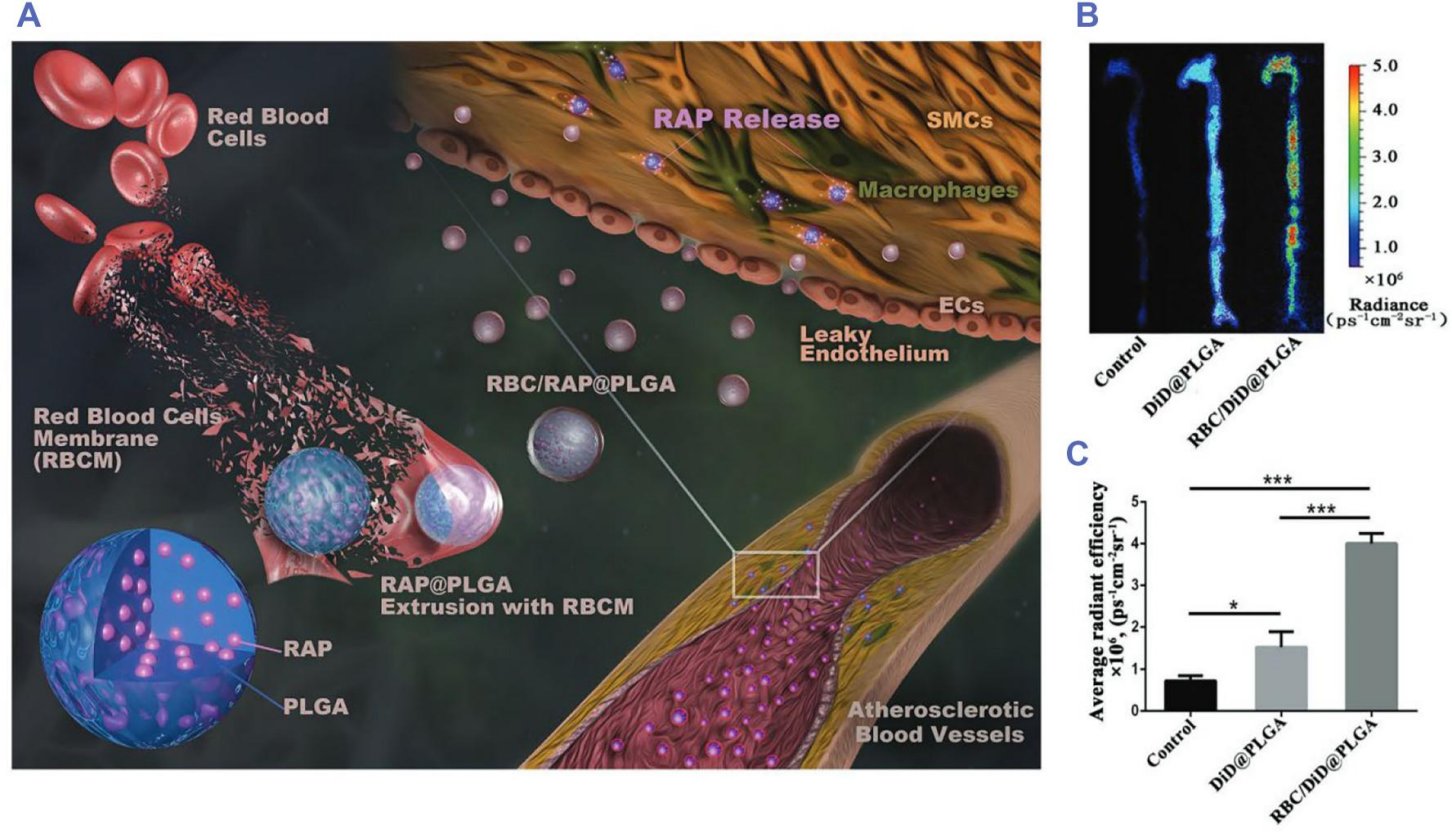
Red blood cell membrane‐based drug delivery system for atherosclerotic therapy. (A) Illustrations displaying the preparation of red blood cell‐coated rapamycin nanoparticles (RBC/RAP@PLGA) for the treatment of atherosclerosis. (B) Ex vivo fluorescence images and (C) quantitative data of fluorescence signals accumulated in the aorta of ApoE^−/−^ mice after injection of 5% sucrose (Control), PLAG nanoparticles loaded with DiD (DiD@PLGA), and red blood cell‐coated DiD nanoparticles (RBC/DiD@PLGA). Reproduced under terms of the CC‐BY license.^110^ Copyright 2019, The Authors, published by John Wiley and Sons.

Taking advantage of EPR effects in atherosclerosis, Yi Wang et al. first prepared PLAG nanoparticles loaded with rapamycin (RAP@PLGA). Then, RAP@PLGA was coated with red blood cell membranes (RBC/RAP @PLGA). Compared with RAP@PLGA, RBC/RAP@PLGA showed better targeting and therapeutic effects without obvious biological side effects in ApoE^−/−^ mice (Figure 8).^110^

## 4 HYBRID MEMBRANE‐COATED BIOMIMETIC NANOPARTICLES

### 4.1 Liposome hybrid with the cell membrane

Liposomes are a nanoscale system that has come of age.^111^ A large number of studies have shown that liposomes can be used to deliver drugs, vaccines, and contrast agents in the body.^112^ Liposomes are composed of single or multiple concentric lipid bilayers in a water chamber and have good biocompatibility and biodegradability.^112^, ^113^, ^114^ Different from biofilm‐drug delivery systems, liposomes have certain advantages, and they can achieve industrial mass production. The preparation process was not difficult, and the source of raw materials was abundant. Due to its nonmembrane proteins, the targeting character was much weaker than that of biofilm drug carriers. Therefore, by fusing with the biofilm in a certain proportion, the nanodrug carrier system prepared in this way can complement the advantages of the two.

**FIGURE 9:**
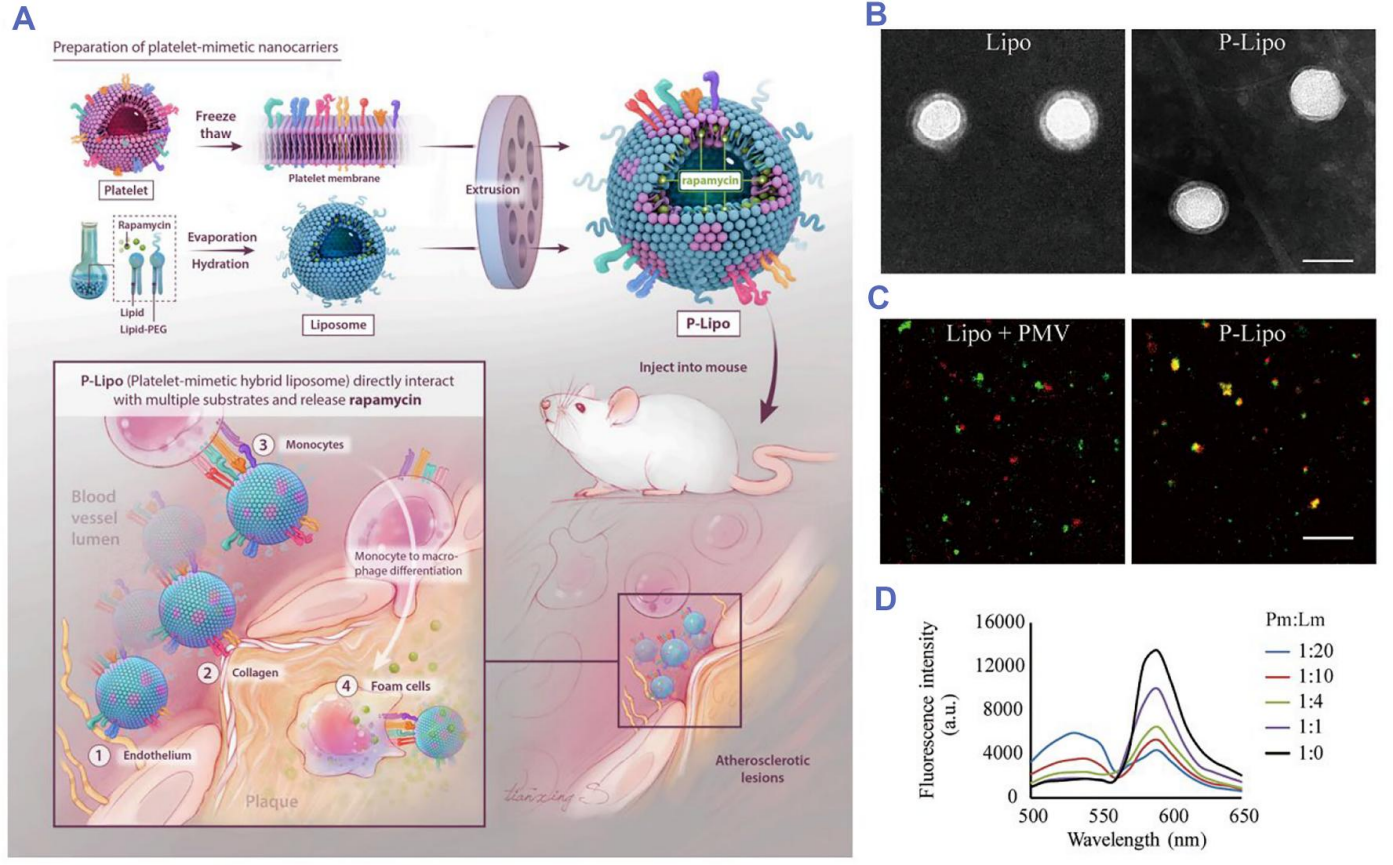
Liposomes hybrid with platelet membrane‐based drug delivery system for atherosclerotic therapy. (A) Schematic of liposome hybrid with P‐Lipo fabrication and targeting treatment for atherosclerosis. (B) TEM of Lipo and P‐Lipo. (C) Confocal fluorescent microscopy images of either a mixture of liposome or PMV or P‐Lipo. (D) PMV was doped with a fluorescence resonance energy transfer (FRET) pair of fluorescent probes and fused with increasing number of liposomes. The recovery of fluorescence emission from the donor at the lower emission peak (536 nm) was monitored (Pm:Lm = platelet membrane superficial area to liposome superficial area ratio). Reproduced with permission.^116^ Copyright 2021, Elsevier. P‐Lipo, platelet membrane‐loaded rapamycin nanoparticle; PMV, platelet membrane vesicle.

A systematic study of hybrid erythrocyte liposomes was reported by Sebastian Himbert et al. in 2020.^115^ The authors fabricated hybrid erythrocyte liposomes by mixing red blood cell membranes with different classes and various saturation degrees of liposomes and proved that the hybrid liposomes could encapsulate small molecules.^115^ In a recent study by Yanan Song et al., a liposome hybrid with a platelet membrane was designed to form a biomimetic nanoparticle (P‐Lipo) for targeting atherosclerotic plaques. As a drug delivery system, P‐Lipo has been proven to have a promising targeting ability and enhance the therapeutic effects of rapamycin in ApoE^−/−^ mice. In addition, P‐Lipo also provides a novel approach for large‐scale production and downstream translation (Figure 9).^116^

### 4.2 Exosomes hybridize with the cell membrane

Exosomes are secretory organelles with a single membrane, and their diameters are approximately 30–200 nm. They can carry and transport specific proteins, lipids, nucleic acids and glycoconjugates.^117^ Exosomes are produced by sprouting on the plasma membrane and endosomal membrane and can transmit messages and molecules to other cells.^118^, ^119^, ^120^ This intercellular vesicle transport pathway plays an important role in many aspects of human health and disease.^121^, ^122^, ^123^, ^124^

**FIGURE 10:**
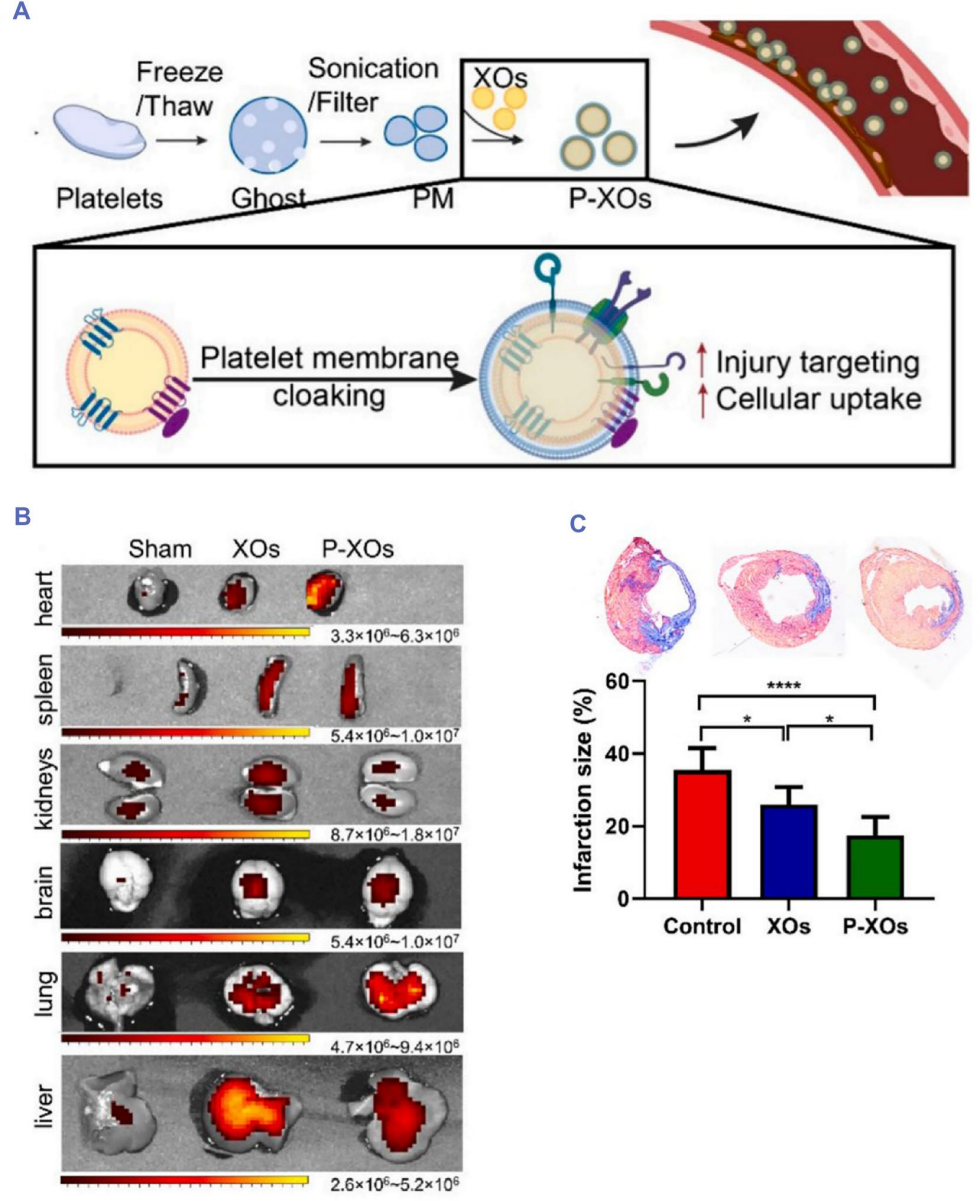
Exosomes hybrid with platelet membrane‐based drug delivery system for cardiovascular disease therapy. (A) Schematic illustrating the preparation of integrated platelet membrane and stem cell exosomes (P‐OXs). (B) Representative ex vivo fluorescence images showing the distribution of stem cell exosomes (XOs) and P‐XOs in the organs of the mouse model of myocardial infarction. Mice were sacrificed on Day 1. (C) Representative Masson trichrome images showing control (PBS‐treated animals), XOs and P‐XOs (from left to right) and the quantification of infarct sizes. Reproduced with permission.^129^ Copyright 2021, Elsevier.

With the in‐depth study of exosomes, in recent years, many innovative nanopreparations have been continuously developed for the diagnosis and treatment of tumors and inflammation based on research on various exosomes.^125^, ^126^ For example, Oshra Betzer et al. labeled mesenchymal stem cell‐derived exosomes with gold nanoparticles (GNPs) for neuroimaging in vivo.^127^ Hongzhao Qi et al. fabricated reticulocyte‐derived exosomes with superparamagnetic Fe_3_O_4_ and antitumor drugs (D‐SMNC‐EXOs), which showed promising imaging and therapeutic effects.^128^ For myocardial injury treatment with exosome hybrid cell membranes, Shiqi Hu et al. first integrated platelet membranes and stem cell exosomes (P‐XOs). Compared with mesenchymal stem cell‐derived exosomes (MSC‐XOs), P‐XOs enhanced cellular uptake by endothelial cells and cardiomyocytes in injured vasculature and improved cardiac function in a myocardial infarction model without obvious toxic side effects.^129^ In atherosclerosis therapy, exosome hybrid cell membranes may become a novel delivery system for atherosclerotic plaques (Figure 10).

## 5 SYNTHETIC NANOPARTICLES

### 5.1 Synthesized nanoparticles with dextran

Dextran is a homopolysaccharide composed of glucose, a type of polymer with branched glucose, and its molecular weight ranges from 1 to 2000 kDa. Because dextran has good stability, biocompatibility, and degradability, it is widely used in the field of biochemistry research.^130^, ^131^, ^132^

#### 5.1.1 Mechanism of targeting to atherosclerotic plaque based on synthesized nanoparticles with dextran

Atherosclerosis is a chronic inflammation, and C‐type lectin plays a vital role in its occurrence and development.^133^ C‐type lectins mainly include versican, DNGR1 (a Dendritic Cell‐Specific sensor of tissue damage) and mincle, which play an important regulatory role in vascular smooth muscle and macrophage proliferation and induce proinflammatory mediators.^133^, ^134^, ^135^ C‐type lectin is often expressed on the surface of macrophages, and dextran can specifically bind to C‐type lectin.^136^ Using this adhesion effect, dextran lays the foundation for targeting atherosclerotic plaques.

#### 5.1.2 Diagnosis of atherosclerosis based on synthesized nanoparticles with dextran

**FIGURE 11:**
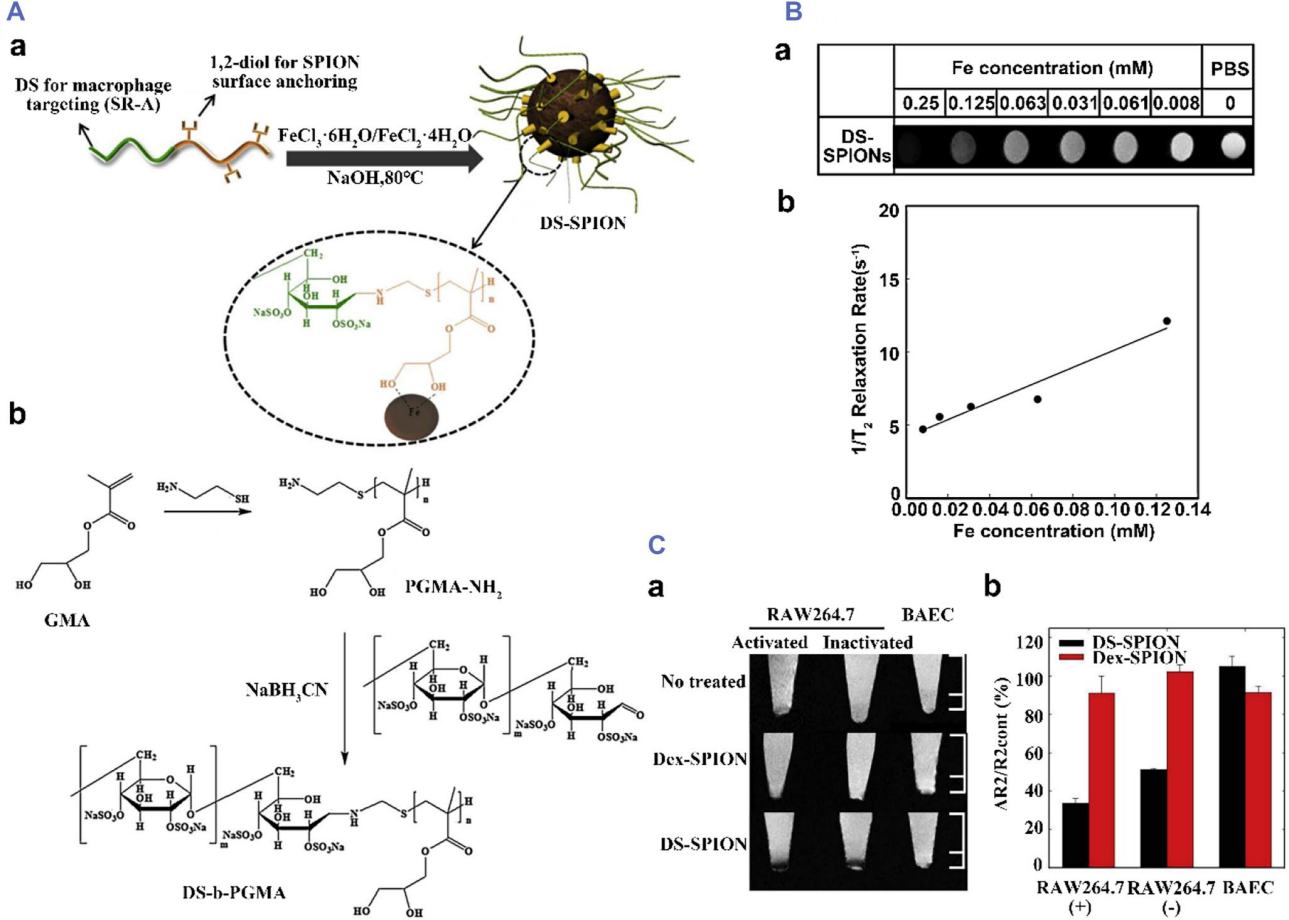
Synthesized nanoparticles with dextran for atherosclerotic diagnosis (A) (a) Illustration of interactions between tailor‐made double hydrophilic DS‐b‐poly (glycerol methacrylate) (DS‐b‐PGMA) and superparamagnetic iron oxide nanoparticles (SPION) and (b) reaction scheme for the synthesis of DS‐b‐PGMA copolymer. (B) (a) T2‐weighted images of DS‐SPIONs at various iron concentrations, (b) T2 relaxation rate (1/T2) as a function of iron concentration for DS‐SPIONs. (C) (a) T2‐weighted MR images and (b) relative signal intensity of RAW264.7 and BAE cells after incubation with nanoparticles. The error bar represents the standard deviation (*n* = 3). Reproduced with permission.^137^ Copyright 2014, Elsevier.

For tracking macrophages, Dong Gil You et al. made further improvements on the basis of dextran‐encapsulated superparamagnetic iron oxide nanoparticles (Dex‐SPIONs). They used a simple coprecipitation method to prepare dextran sulfate‐coated superparamagnetic iron oxide nanoparticles (DS‐SPIONs). T2‐weighted MRI of the cells showed that the activated macrophages treated with DS‐SPIONs showed significantly lower signal intensity than those treated with Dex‐SPIONs.^137^ To target atherosclerotic plaques in vivo, Keiko Tsuchiya et al. conducted experiments in a rabbit atherosclerotic model. Dextran and mannan‐dextran were used to coat USPIO to prepare D‐USPIO and DM‐USPIO. MRI scans showed that DM‐USPIO is better than D‐USPIO in the study of atherosclerotic lesions in rabbits.^138^ These results show that magnetic IONPs coated with dextran have a good targeting effect. Based on the modifiable properties of dextran, some specially modified dextran‐coated magnetic IONPs have better imaging effects. They could be used as potential MRI contrast agents (Figure 11).^137^

#### 5.1.3 Treatment of atherosclerosis based on synthesized nanoparticles with dextran

**FIGURE 12:**
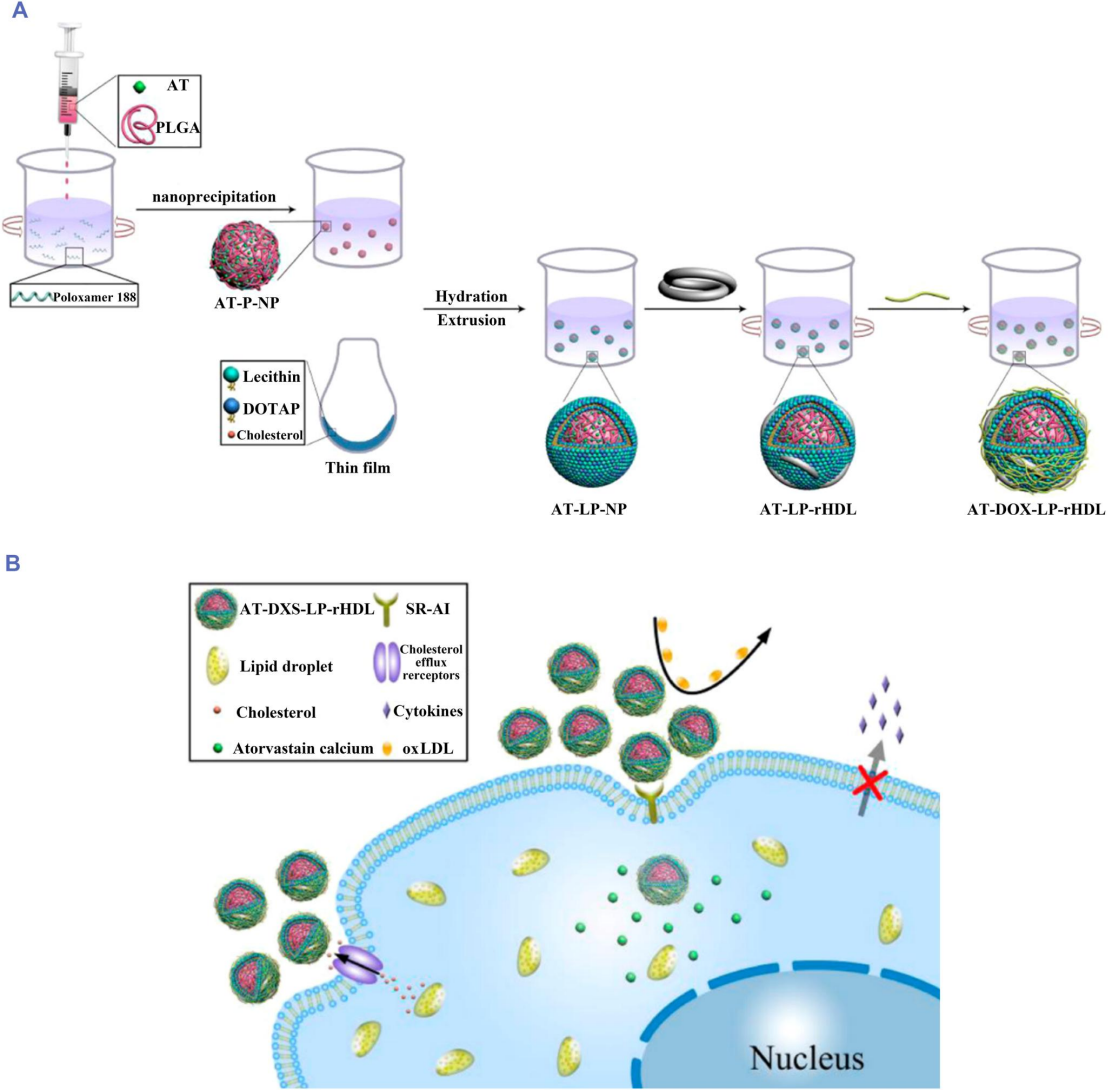
Synthesized nanoparticles with dextran for atherosclerotic therapy. (A) Schematic diagram of the preparation procedures of atorvastatin calcium (AT)‐loaded dextran sulfate (DXS)‐coated core−shell reconstituted high‐density lipoprotein (rHDL), which is referred to as AT‐DXS‐L. (B) Schematic illustration of the targeted delivery of AT‐DXS‐L. Reproduced with permission.^139^ Copyright 2017, American Chemical Society.

For atherosclerosis therapy with dextran‐coated nanoparticles, Yi Zhao et al. first prepared AT‐DXS‐LP‐rHDL, which was an atorvastatin calcium (AT) coated with dextran sulfate (DXS), and the core‐shell was also reconstituted with high‐density lipoprotein (rHDL). Via DXS with high affinity for scavenging receptor‐like AI (SR‐AI), the nanoparticles could deliver drugs to foam cells, and inflammation could be inhibited by depleting intracellular cholesterol through apolipoprotein A‐I (apoA‐I)‐mediated cholesterol efflux.^139^ Overall, AT‐DXS‐LP‐rHDL, as a multifunctional carrier, can not only deliver more drugs to macrophages but also inhibit the synthesis of oxidized low‐density lipoprotein (oxLDL) and promote cholesterol efflux, indicating the antiatherosclerotic effect of the biofunctional nanocarriers (Figure 12).

### 5.2 Synthesized nanoparticles with antibodies

Because antibodies can bind to a specific protein, this confers antibody with the ability to target. Taking advantage of this property, many nanoparticles synthesized by antibodies are widely used in research.

#### 5.2.1 Mechanism of targeting to atherosclerotic plaque based on synthesized nanoparticles with antibodies

During the development of atherosclerosis, inflammatory cells such as endothelial cells and macrophages are activated at the site of inflammation and highly express specific proteins. However, these proteins were expressed little or not at all in normal tissues. Therefore, taking advantage of the specific expression of these inflammatory proteins in the inflammatory site or inflammatory cells, inflammatory protein antibodies were used to synthesize nanoparticles, which were designed to encapsulate imaging molecules or therapeutic drugs for the diagnosis or treatment of atherosclerosis.

#### 5.2.2 Diagnosis of atherosclerosis based on synthesized nanoparticles with antibodies

**FIGURE 13:**
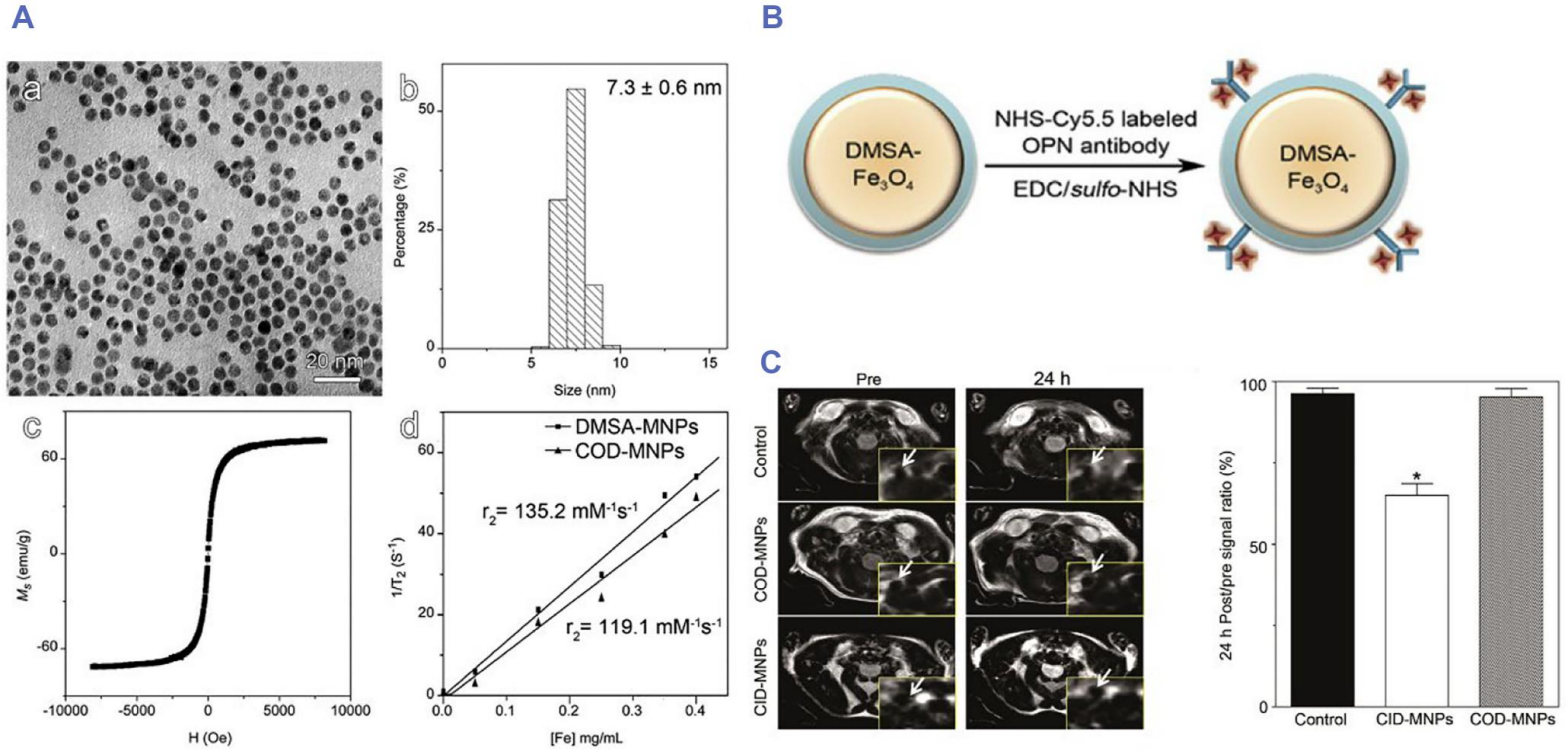
Synthesized nanoparticles with antibody for atherosclerotic diagnosis (A) TEM image (a), the histogram of particle size (b), saturation magnetization curve (c) of DMSA‐coated Fe_3_O_4_ magnetic nanoparticles (DMSA‐MNPs), together with 1/T2 plotted against the concentration of Fe (d) for extracting relaxivity (r2) of DMSA‐MNPs and the Cy5.5‐OPN‐DMSA‐coated magnetic nanoparticles (COD‐MNPs) as inserted. (B) Hydrodynamic profiles of the DMSA‐MNPs (NP) and the COD‐MNPs conjugates (NP‐OPN). (C) MR images of HFD‐fed mice acquired at different time points after intravenous injection of different probes for comparison with those that received no probe injection. The right part of the panel shows the quantitative in vivo T2 signal of the region of interest after intravenous injection of COD‐MNPs or CID‐MNPs, *p < 0.05. Reproduced with permission.^141^ Copyright 2017, Elsevier.

It has been reported that osteopontin (OPN) is highly expressed in foam cells.^140^ Since foam cells have been identified as an important component of vulnerable atherosclerotic lesions, OPN may become a potential target for vulnerable atherosclerotic plaque imaging. Hongyu Qiao et al. designed Cy5.5‐OPN‐DMSA‐MNPs (COD‐MNPs), and the core of these nanoparticles was Fe_3_O_4_ magnetic nanoparticles (MNPs). The shell was composed of meso‐2,3‐Dimercaptosuccinic acid (DMSA), and the OPN antibody labeled with Cy5.5 was attached to the surface of the DMSA shell. This nanoparticle enables not only fluorescence imaging but also MRI. In vitro cell experiments showed that the COD‐MNPs could specifically recognize foam cells. In vivo animal model studies have demonstrated that the visualization of vulnerable plaques can be achieved by intravenous injection of COD‐MNPs.^141^ The author's study successfully synthesized antibodies on nanoparticles, which enabled the Fe_3_O_4_‐loaded nanoparticles to be effectively targeted to unstable plaques and provided a new strategy for non‐invasive atherosclerosis imaging (Figure 13).

#### 5.2.3 Treatment of atherosclerosis based on synthesized nanoparticles with antibodies

**FIGURE 14:**
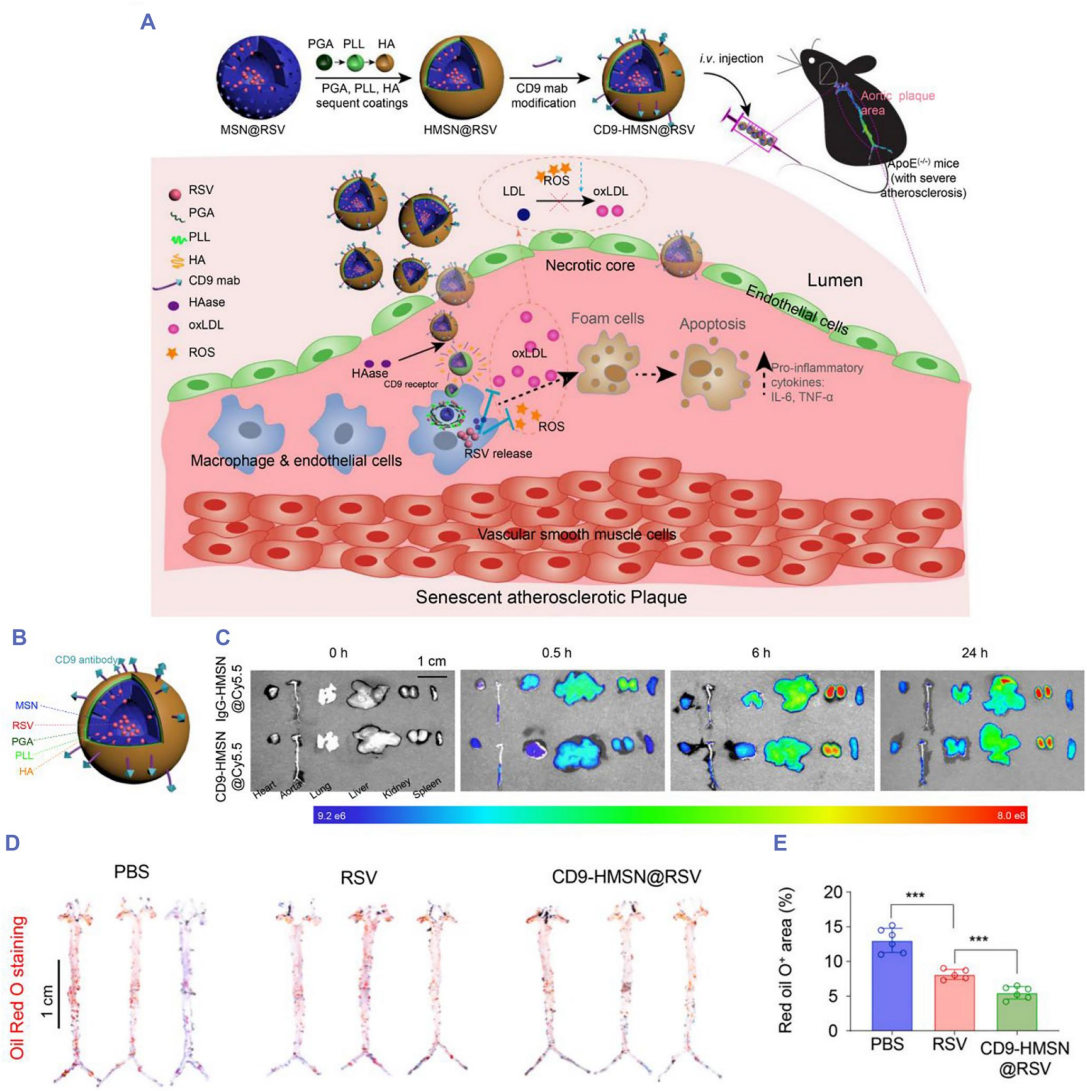
Synthesized nanoparticles with antibodies for atherosclerotic therapy. (A) Schematic representation of CD9‐HMSN@RSV, in which the antisenescence drug rosuvastatin (RSV) was loaded in CD9‐modified MSNs coated with hyaluronic acid (HA), poly(L‐lysine hydrochloride) (PLL), and methoxy‐poly(ethylene glycol)‐block‐poly(L‐glutamic acid sodium salt) (PGA). The CD9‐HMSN@RSV nanoparticles mitigated atherosclerosis via selective targeting and clearance of senescent cells in atherosclerotic plaques with high CD9 expression. (B) Schematic illustration of nanoparticles coated with different layers. (C) By labeling the nanoparticles with Cy5.5 to form IgG‐HMSN@Cy5.5 and CD9‐HMSN@Cy5.5 and ex vivo distribution of IgG‐HMSN@Cy5.5 and CD9‐ HMSN@Cy5.5 in different organs (including the heart, aorta, lung, liver, kidney, and spleen) isolated from ApoE^−/−^ mice at 0, 0.5, 6, and 24 h after intravenous injection. (D) Three representative images of isolated aortas stained with Oil red O following treatment with PBS, RSV, and CD9‐HMSN@RSV. (E) Quantitative analysis of areas with Oil red O staining (*n* = 6). ***p < 0.001. Reproduced with permission.^143^ Copyright 2021, Elsevier.

Cluster of differentiation 9 (CD9), as a cell surface glycoprotein, was found to be highly expressed in human aortas and coronary arteries, especially in areas of atherosclerosis.^142^ For atherosclerosis therapy, Le Minh Pham et al. designed a drug delivery system termed CD9‐HSMN@RSV. The core of the system was mesoporous silica nanoparticles (MSNs) loaded with rosuvastatin (RSV). The shell of the system was constructed with hyaluronic acid (HA), poly (L‐lysine hydrochloride) (PLL), and methoxy‐poly (ethylene glycol)‐block‐poly (L‐glutamic acid sodium salt) (PGA), and CD9 was embedded in the surface of the shell. In vivo and in vitro, CD9‐HSMN@RSV showed good targeting ability to efficiently transport RSV into foam cells (in vivo) and atherosclerotic plaques (in vitro), which effectively reduced the production of ROS, attenuated the oxidation of LDL and alleviated the secretion of proinflammatory cytokines.^143^ This study provides a deeper look at the cellular markers expressed by senescent cells in aortic plaques and may imply a more precise and effective strategy to target and mitigate atherosclerosis (Figure 14).

## 6 CONCLUSION AND PERSPECTIVES

Currently, with the understanding of the dynamic biological functions of cells, various types of cells have been developed in delivery systems for targeted therapeutic applications, including the diagnosis or treatment of autoimmune diseases, cancer and infectious diseases. Compared with traditional diagnostic or therapeutic strategies, the combination of immunotherapy and cell therapy has achieved greater clinical success.

With the in‐depth study of the pathogenesis of atherosclerosis, diagnostic methods and treatment strategies are also undergoing innovation. Membrane‐based nanotherapeutics combine the intrinsic properties of cells and the functional diversity of nanomaterials to deliver therapeutics with tissue‐targeted precision, providing a new strategy for the diagnosis and treatment of atherosclerosis. Unlike traditional treatment regimens, native cell membranes have the ability to target through interactions between the surface components of native cell membranes and target cells, so cell membranes have been used for drug delivery (e.g., rapamycin and statins) to target atherosclerosis or carry nanoparticles (e.g., Fe_3_O_4_ nanoparticles) to noninvasively locate the site of atherosclerotic plaque by MRI (Table 1).

**TABLE 1:** Summary of biomimetic nanoparticles targeting atherosclerosis for diagnosis and therapy.

Source	Nanoparticle platform	Active pharmaceutical ingredients	Application	Target (protein or cells)	Refs
Macrophages 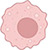	Macrophage membrane‐coated nanoparticles	Fe_3_O_4_	MRI	VCAM‐1	75
	Macrophage membrane‐coated nanoparticles	Fe_3_O_4_	MRI	Osteopontin	141
	Macrophage membrane‐coated nanoparticles	RAP	Therapy	VCAM‐1, SIRPα‐R	76
	Modified macrophage membrane‐coated nanoparticles	Colchicine	Therapy	VCAM‐1, SIRPα‐R	144
Platelets 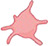	Platelets membrane coated nanoparticles	Gadolinium	MRI	Collagen, macrophage	91
	Platelets membrane coated nanoparticles	DiD	Fluorescent images	vWF, collagen, fibrin	145
	Platelets membrane coated MSN	CD47	Therapy	Collagen, macrophage	92
Red blood cells 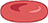	Red blood cells membrane coated nanoparticles	RAP	Therapy	Leaky endothelium	110
Hybrid membrane 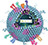	Liposome hybrid with the cell membrane nano‐vessels	RAP	Therapy	vWF, collagen	116
	Exosome hybrid with the platelet membrane nano‐vessels	Stem cell exosomes	Therapy	Myocardial infarction	129
Synthetic materials 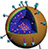	Dextran sulfate‐coated superparamagnetic iron oxide nanoparticles	USPIO	MRI	Foam cells	137
	Atorvastatin calcium‐loaded dextran sulfate—coated core—shell reconstituted high‐density lipoprotein	Atorvastatin calcium	Therapy	Foam cells	139
	OPN antibody attached to DMSA coated Fe3O4 nanoparticles	Fe_3_O_4_ magnetic nanoparticles	MRI	Osteopontin	141
	CD9 antibody attached to HA coated MSN nanoparticles loaded with rosuvastatin	CD9 antibody	Therapy	Rosuvastatin	143

Breakthroughs have also been made in hybrid membrane delivery systems derived from cell membranes. Hybridization and fusion of liposomes or exosome membranes with different cell membranes can effectively improve the utilization of cell membranes and enhance targeting characteristics. Although they also have certain limitations, such as the relatively cumbersome preparation process and the stability to be further improved, this hybrid fusion membrane still has huge research and development space and therapeutic potential.

For the drug‐delivery system prepared by synthetic membranes, some specific antibodies are artificially embedded on their surfaces so that these antibodies can be effectively targeted to the atherosclerotic plaque site. This finding also provides new avenues for targeting atherosclerotic plaques. Since there are far more types of antibodies than cell membranes, and antibodies can more precisely bind to specific proteins, this also provides more ideas and strategies for the preparation of nanodrug delivery systems.

Recently, Hydrogel polymeric materials are also gradually used in the treatment of atherosclerosis.^146^ Unlike traditional intravenous nanomaterials, hydrogels are subcutaneous injections of drug‐loaded hydrogels to regulate cholesterol efflux and inflammation to inhibit the pathological progression of atherosclerosis, which may help develop a promising nanomedicine.

For targeted therapy strategies, Chimeric Antigen Receptor T‐Cell Immunotherapy (CAR‐T) therapy is in the spotlight.^147^ Although its clinical application has made breakthroughs,^148^, ^149^ it is difficult to be widely used in clinical practice due to its technical difficulty and extremely high cost. The development and preparation of nanomaterials provide an updated strategy and effective method for targeted therapy.

Overall, in this review, we highlight the importance of cell membrane‐based delivery systems in atherosclerosis diagnostic and therapeutic applications and underscore the importance of multidisciplinary integration, which indicates the great potential for clinical application with biomimetic nano‐delivery systems.

## AUTHOR CONTRIBUTIONS

Yuyu Li: Conceptualization; software; data curation; writing – original draft; visualization. Jifang Wang: Conceptualization; data curation; writing – original draft; visualization. Jun Xie: Writing – review and editing; supervision; project administration; funding acquisition.

## CONFLICT OF INTEREST STATEMENT

All authors declare that there are no competing interests.

## References

[1] A. Gallino , V. Aboyans , C. Diehm , F. Cosentino , H. Stricker , E. Falk , O. Schouten , J. Lekakis , B. Amann‐Vesti , F. Siclari , P. Poredos , S. Novo , M. Brodmann , K. Schulte , C. Vlachopoulos , R. De Caterina , P. Libby , I. Baumgartner , Eur. Heart J. 2014, 35, 1112. 24595865 10.1093/eurheartj/ehu071

[2] D. Capodanno , M. Alberts , D. Angiolillo , Nat. Rev. Cardiol. 2016, 13, 609. 27489191 10.1038/nrcardio.2016.111

[3] R. Corti , V. Fuster , Eur. Heart J. 2011, 32, 1709. 21508002 10.1093/eurheartj/ehr068

[4] A. Gisterå , G. Hansson , Nat. Rev. Nephrol. 2017, 13, 368. 28392564 10.1038/nrneph.2017.51

[5] R. Ross , N. Engl. J. Med. 1999, 340, 115. 9887164 10.1056/NEJM199901143400207

[6] P. Libby , Nature 2002, 420, 868. 12490960 10.1038/nature01323

[7] A. Usman , D. Ribatti , U. Sadat , J. Gillard , J. Atheroscler. Thromb. 2015, 22, 739. 26156748 10.5551/jat.30460

[8] P. Shah , S. Bajaj , H. Virk , M. Bikkina , F. Shamoon , Thrombosis 2015, 2015, 634983. 26823982 10.1155/2015/634983 PMC4707354

[9] T. Zhao , Z. Mallat , J. Am. Coll. Cardiol. 2019, 73, 1691. 30947923 10.1016/j.jacc.2018.12.083

[10] G. Tumurkhuu , J. Dagvadorj , R. Porritt , T. Crother , K. Shimada , E. Tarling , E. Erbay , M. Arditi , S. Chen , Cell Metab. 2018, 28, 432. 29937375 10.1016/j.cmet.2018.05.027 PMC6125162

[11] N. Pothineni , S. Subramany , K. Kuriakose , L. Shirazi , F. Romeo , P. Shah , J. Mehta , Eur. Heart J. 2017, 38, 3195. 29020241 10.1093/eurheartj/ehx362

[12] A. Jackson , M. Regine , C. Subrata , S. Long , IJC Heart Vasc. 2018, 21, 36. 10.1016/j.ijcha.2018.09.006 PMC6161413 30276232

[13] T. Gerhardt , K. Ley , Cardiovasc. Res. 2015, 107, 321. 25990461 10.1093/cvr/cvv147 PMC4592323

[14] I. Tabas , A. Lichtman , Immunity 2017, 47, 621. 29045897 10.1016/j.immuni.2017.09.008 PMC5747297

[15] F. Tacke , D. Alvarez , T. Kaplan , C. Jakubzick , R. Spanbroek , J. Llodra , A. Garin , J. Liu , M. Mack , N. van Rooijen , S. Lira , A. Habenicht , G. Randolph , J. Clin. Invest. 2007, 117, 185. 17200718 10.1172/JCI28549 PMC1716202

[16] G. Koelwyn , E. Corr , E. Erbay , K. Moore , Nat. Immunol. 2018, 19, 526. 29777212 10.1038/s41590-018-0113-3 PMC6314674

[17] A. Sage , D. Tsiantoulas , C. Binder , Z. Mallat , Nat. Rev. Cardiol. 2019, 16, 180. 30410107 10.1038/s41569-018-0106-9

[18] G. Hansson , A. Hermansson , Nat. Immunol. 2011, 12, 204. 21321594 10.1038/ni.2001

[19] D. Ketelhuth , G. Hansson , Circ. Res. 2016, 118, 668. 26892965 10.1161/CIRCRESAHA.115.306427

[20] T. Seijkens , K. Poels , S. Meiler , C. van Tiel , P. Kusters , M. Reiche , D. Atzler , H. Winkels , M. Tjwa , H. Poelman , B. Slütter , J. Kuiper , M. Gijbels , J. Kuivenhoven , L. Matic , G. Paulsson‐Berne , U. Hedin , G. Hansson , G. Nicolaes , M. Daemen , C. Weber , N. Gerdes , M. de Winther , E. Lutgens , Eur. Heart J. 2019, 40, 372. 30452556 10.1093/eurheartj/ehy714 PMC6340101

[21] R. Saigusa , H. Winkels , K. Ley , Nat. Rev. Cardiol. 2020, 17, 387. 32203286 10.1038/s41569-020-0352-5 PMC7872210

[22] P. Libby , A. Lichtman , G. Hansson , Immunity 2013, 38, 1092. 23809160 10.1016/j.immuni.2013.06.009 PMC3764500

[23] M. Nus , Z. Mallat , Expert Rev. Clin. Immunol. 2016, 12, 1217. 27253721 10.1080/1744666X.2016.1195686

[24] S. Soares , J. Sousa , A. Pais , C. Vitorino , Front. Chem. 2018, 6, 360. 30177965 10.3389/fchem.2018.00360 PMC6109690

[25] K. Greish , A. Mathur , M. Bakhiet , S. Taurin , Ther. Delivery 2018, 9, 269. 10.4155/tde-2017-0118 29495928

[26] J. Cao , D. Huang , N. Peppas , Adv. Drug Delivery Rev. 2020, 167, 170. 10.1016/j.addr.2020.06.030 32622022

[27] E. Blanco , H. Shen , M. Ferrari , Nat. Biotechnol. 2015, 33, 941. 26348965 10.1038/nbt.3330 PMC4978509

[28] W. G. Kreyling , M. Semmler‐Behnke , Q. Chaudhry , Nano Today 2010, 5, 165.

[29] J. Siegel , M. Polivkova , M. Staszek , K. Kolarova , S. Rimpelova , V. Svorcik , Mater. Lett. 2015, 145, 87.

[30] M. Polivkova , M. Valova , S. Rimpelova , P. Slepicka , V. Svorcik , J. Siegel , Express Polym. Lett. 2018, 12, 1039.

[31] M. Staszek , J. Siegel , S. Rimpelova , O. Lyutakov , V. Svorcik , Mater. Lett. 2015, 158, 351.

[32] S. Coiai , E. Passaglia , A. Pucci , G. Ruggeri , Materials 2015, 8, 3377.

[33] X. Zheng , P. Zhang , Z. Fu , S. Meng , L. Dai , H. Yang , RSC Adv. 2021, 11, 19041. 35478636 10.1039/d1ra01849c PMC9033557

[34] M. Fathi‐Achachelouei , H. Knopf‐Marques , C. E. Ribeiro da Silva , J. Barthes , E. Bat , A. Tezcaner , N. E. Vrana , Front. Bioeng. Biotechnol. 2019, 7, 113. 10.3389/fbioe.2019.00113 PMC6543169 31179276

[35] Y. Yang , S. Wang , Y. Wang , X. Wang , Q. Wang , M. Chen , Biotechnol. Adv. 2014, 32, 1301. 25109677 10.1016/j.biotechadv.2014.07.007

[36] S. K. Bhunia , A. Saha , A. R. Maity , S. C. Ray , N. R. Jana , Sci. Rep. 2013, 3, 1473. 23502324 10.1038/srep01473 PMC3600594

[37] E. Keles , Y. Song , D. Du , W. Dong , Y. Lin , Biomater. Sci. 2016, 4, 1291. 27480033 10.1039/c6bm00441e PMC13102266

[38] A. C. Gomes , M. Mohsen , M. F. Bachmann , Vaccines 2017, 5, 6. 28216554 10.3390/vaccines5010006 PMC5371742

[39] D. Kim , J. Kim , Y. I. Park , N. Lee , T. Hyeon , ACS Cent. Sci. 2018, 4, 324. 29632878 10.1021/acscentsci.7b00574 PMC5879478

[40] M. Vedhanayagam , A. S. Kumar , B. U. Nair , K. J. Sreeram , Appl. Biochem. Biotechnol. 2022, 194, 266. 34817807 10.1007/s12010-021-03764-w

[41] A. A. Yetisgin , S. Cetinel , M. Zuvin , A. Kosar , O. Kutlu , Molecules 2020, 25, 2193. 32397080 10.3390/molecules25092193 PMC7248934

[42] A. P. Dias , S. da Silva Santos , J. V. da Silva , R. Parise‐Filho , E. I. Ferreira , O. El Seoud , J. Giarolla , Int. J. Pharm. 2020, 573, 118814. 31759101 10.1016/j.ijpharm.2019.118814

[43] X. Wang , L. Liu , O. Ramstroem , M. Yan , Exp. Biol. Med. 2009, 234, 1128. 10.3181/0904-MR-134 PMC4037294 19596820

[44] M. Alshamrani , Polymers 2022, 14, 1221. 35335551 10.3390/polym14061221 PMC8956086

[45] M. Gifani , D. J. Eddins , H. Kosuge , Y. Zhang , S. L. A. Paluri , T. Larson , N. Leeper , L. A. Herzenberg , S. S. Gambhir , M. V. McConnell , E. E. B. Ghosn , B. R. Smith , Adv. Funct. Mater. 2021, 31, 2101005. 34733130 10.1002/adfm.202101005 PMC8559995

[46] A. M. Flores , N. Hosseini‐Nassab , K. U. Jarr , J. Ye , X. Zhu , R. Wirka , A. L. Koh , P. Tsantilas , Y. Wang , V. Nanda , Y. Kojima , Y. Zeng , M. Lotfi , R. Sinclair , I. L. Weissman , E. Ingelsson , B. R. Smith , N. J. Leeper , Nat. Nanotechnol. 2020, 15, 154. 31988506 10.1038/s41565-019-0619-3 PMC7254969

[47] P. Falcaro , R. Ricco , A. Yazdi , I. Imaz , S. Furukawa , D. Maspoch , R. Ameloot , J. D. Evans , C. J. Doonan , Coord. Chem. Rev. 2016, 307, 237.

[48] M. E. Kooi , V. C. Cappendijk , K. Cleutjens , A. G. H. Kessels , P. Kitslaar , M. Borgers , P. M. Frederik , M. Daemen , J. M. A. van Engelshoven , Circulation 2003, 107, 2453. 12719280 10.1161/01.CIR.0000068315.98705.CC

[49] K. A. Kelly , J. R. Allport , A. Tsourkas , V. R. Shinde‐Patil , L. Josephson , R. Weissleder , Circ. Res. 2005, 96, 327. 15653572 10.1161/01.RES.0000155722.17881.dd

[50] M. Nahrendorf , F. A. Jaffer , K. A. Kelly , D. E. Sosnovik , E. Aikawa , P. Libby , R. Weissleder , Circulation 2006, 114, 1504. 17000904 10.1161/CIRCULATIONAHA.106.646380

[51] P. W. K. Rothemund , N. Papadakis , E. Winfree , PLoS Biol. 2004, 2, 2041. 10.1371/journal.pbio.0020424 PMC534809 15583715

[52] P. W. K. Rothemund , Nature 2006, 440, 297. 16541064 10.1038/nature04586

[53] H. Li , T. H. LaBean , K. W. Leong , Interface Focus 2011, 1, 702. 23050076 10.1098/rsfs.2011.0040 PMC3262286

[54] E. S. Andersen , M. Dong , M. M. Nielsen , K. Jahn , R. Subramani , W. Mamdouh , M. M. Golas , B. Sander , H. Stark , C. L. P. Oliveira , J. S. Pedersen , V. Birkedal , F. Besenbacher , K. V. Gothelf , J. Kjems , Nature 2009, 459, 73. 19424153 10.1038/nature07971

[55] S. M. Douglas , H. Dietz , T. Liedl , B. Hogberg , F. Graf , W. M. Shih , Nature 2009, 459, 414. 19458720 10.1038/nature08016 PMC2688462

[56] S. H. Ko , M. Su , C. Zhang , A. E. Ribbe , W. Jiang , C. Mao , Nat. Chem. 2010, 2, 1050. 21107369 10.1038/nchem.890 PMC3059202

[57] Y. Gao , X. Chen , T. Tian , T. Zhang , S. Gao , X. Zhang , Y. Yao , Y. Lin , X. Cai , Adv. Mater. 2022, 34, 2201731. 10.1002/adma.202201731 35511782

[58] X. Liu , D. Duan , Y. Wang , J. Liu , D. Duan , Front. Biosci. 2022, 27, 61. 10.31083/j.fbl2702061 35227004

[59] I. L. Aanei , T. Huynh , Y. Seo , M. B. Francis , Bioconjugate Chem. 2018, 29, 2526. 10.1021/acs.bioconjchem.8b00453 30059611

[60] A. Broisat , S. Hernot , J. Toczek , J. De Vos , L. M. Riou , S. Martin , M. Ahmadi , N. Thielens , U. Wernery , V. Caveliers , S. Muyldermans , T. Lahoutte , D. Fagret , C. Ghezzi , N. Devoogdt , Circ. Res. 2012, 110, 927. 22461363 10.1161/CIRCRESAHA.112.265140 PMC3918224

[61] A. O. Elzoghby , W. M. Samy , N. A. Elgindy , J. Controlled Release 2012, 157, 168. 10.1016/j.jconrel.2011.07.031 21839127

[62] Y. Luo , Y. Guo , H. Wang , M. Yu , K. Hong , D. Li , R. Li , B. Wen , D. Hu , L. Chang , J. Zhang , B. Yang , D. Sun , A. S. Schwendeman , Y. Eugene Chen , EBioMedicine 2021, 74, 103725. 34879325 10.1016/j.ebiom.2021.103725 PMC8654800

[63] L. Di , A. Maiseyeu , Drug Delivery 2021, 28, 408. 33594923 10.1080/10717544.2021.1886199 PMC7894439

[64] F. F. Davis , Adv. Drug Delivery Rev. 2002, 54, 457. 10.1016/s0169-409x(02)00021-2 12052708

[65] J. V. Jokerst , T. Lobovkina , R. N. Zare , S. S. Gambhir , Nanomedicine 2011, 6, 715. 21718180 10.2217/nnm.11.19 PMC3217316

[66] G. Gangapurwala , A. Vollrath , A. De San Luis , U. S. Schubert , Pharmaceutics 2020, 12, 1118. 33233637 10.3390/pharmaceutics12111118 PMC7699691

[67] P. Gentile , V. Chiono , I. Carmagnola , P. V. Hatton , Int. J. Mol. Sci. 2014, 15, 3640. 24590126 10.3390/ijms15033640 PMC3975359

[68] M. J. Jacobin‐Valat , J. Laroche‐Traineau , M. Lariviere , S. Mornet , S. Sanchez , M. Biran , C. Lebaron , J. Boudon , S. Lacomme , M. Cerutti , G. Clofent‐Sanchez , Nanomed. Nanotechnol. Biol. Med. 2015, 11, 927. 10.1016/j.nano.2014.12.006 25684334

[69] E. F. Craparo , M. Cabibbo , A. Conigliaro , M. M. Barreca , T. Musumeci , G. Giammona , G. Cavallaro , Pharmaceutics 2021, 13, 503. 33916918 10.3390/pharmaceutics13040503 PMC8067637

[70] F. McWhorter , C. Davis , W. Liu , Cell Mol. Life Sci. 2015, 72, 1303. 25504084 10.1007/s00018-014-1796-8 PMC4795453

[71] P. Murray , T. Wynn , Nat. Rev. Immunol. 2011, 11, 723. 21997792 10.1038/nri3073 PMC3422549

[72] T. Tang‐Huau , P. Gueguen , C. Goudot , M. Durand , M. Bohec , S. Baulande , B. Pasquier , S. Amigorena , E. Segura , Nat. Commun. 2018, 9, 2570. 29967419 10.1038/s41467-018-04985-0 PMC6028641

[73] T. Wynn , A. Chawla , J. Pollard , Nature 2013, 496, 445. 23619691 10.1038/nature12034 PMC3725458

[74] A. Haghikia , F. Zimmermann , P. Schumann , A. Jasina , J. Roessler , D. Schmidt , P. Heinze , J. Kaisler , V. Nageswaran , A. Aigner , U. Ceglarek , R. Cineus , A. Hegazy , E. van der Vorst , Y. Döring , C. Strauch , I. Nemet , V. Tremaroli , C. Dwibedi , N. Kränkel , D. Leistner , M. Heimesaat , S. Bereswill , G. Rauch , U. Seeland , O. Soehnlein , D. Müller , R. Gold , F. Bäckhed , S. Hazen , A. Haghikia , U. Landmesser , Eur. Heart J. 2022, 43, 518. 34597388 10.1093/eurheartj/ehab644 PMC9097250

[75] X. Huang , C. Lin , C. Luo , Y. Guo , J. Li , Y. Wang , J. Xu , Y. Zhang , H. Wang , Z. Liu , B. Chen , Nanomed. Nanotechnol. Biol. Med. 2021, 33, 102348. 10.1016/j.nano.2020.102348 33321215

[76] Y. Wang , K. Zhang , T. Li , A. Maruf , X. Qin , L. Luo , Y. Zhong , J. Qiu , S. McGinty , G. Pontrelli , X. Liao , W. Wu , G. Wang , Theranostics 2021, 11, 164. 33391468 10.7150/thno.47841 PMC7681077

[77] M. Rodrigues , N. Kosaric , C. Bonham , G. Gurtner , Physiol. Rev. 2019, 99, 665. 30475656 10.1152/physrev.00067.2017 PMC6442927

[78] M. Koupenova , B. Kehrel , H. Corkrey , J. Freedman , Eur. Heart J. 2017, 38, 785. 28039338 10.1093/eurheartj/ehw550 PMC11110018

[79] H. Weiss , N. Engl. J. Med. 1975, 293, 531. 168489 10.1056/NEJM197509112931105

[80] M. Labelle , S. Begum , R. Hynes , Cancer Cell 2011, 20, 576. 22094253 10.1016/j.ccr.2011.09.009 PMC3487108

[81] E. Middleton , A. Weyrich , G. Zimmerman , Physiol. Rev. 2016, 96, 1211. 27489307 10.1152/physrev.00038.2015 PMC6345245

[82] F. Cognasse , S. Laradi , P. Berthelot , T. Bourlet , H. Marotte , P. Mismetti , O. Garraud , H. Hamzeh‐Cognasse , Front. Immunol. 2019, 10, 1478. 31316518 10.3389/fimmu.2019.01478 PMC6611140

[83] F. Gaertner , S. Massberg , Nat. Rev. Immunol. 2019, 19, 747. 31409920 10.1038/s41577-019-0202-z

[84] M. Gawaz , K. Stellos , H. Langer , J. Thromb. Haemost. 2008, 6, 235. 18088342 10.1111/j.1538-7836.2008.02867.x

[85] H. Langer , M. Gawaz , Thromb. Haemost. 2008, 99, 480. 18327395 10.1160/TH07-11-0685

[86] M. Wu , T. Atkinson , J. Lindner , Blood 2017, 129, 1415. 28174163 10.1182/blood-2016-07-692673 PMC5356449

[87] Y. Huo , A. Schober , S. Forlow , D. Smith , M. Hyman , S. Jung , D. Littman , C. Weber , K. Ley , Nat. Med. 2003, 9, 61. 12483207 10.1038/nm810

[88] J. Blanks , T. Moll , R. Eytner , D. Vestweber , Eur. J. Immunol. 1998, 28, 433. 9521050 10.1002/(SICI)1521-4141(199802)28:02<433::AID-IMMU433>3.0.CO;2-U

[89] L. Totani , V. Evangelista , Arterioscler. Thromb. Vasc. Biol. 2010, 30, 2357. 21071701 10.1161/ATVBAHA.110.207480 PMC3076621

[90] P. Kuijper , H. Gallardo Tores , J. Lammers , J. Sixma , L. Koenderman , J. Zwaginga , Thromb. Haemost. 1998, 80, 443. 9759625

[91] X. Wei , M. Ying , D. Dehaini , Y. Su , A. Kroll , J. Zhou , W. Gao , R. Fang , S. Chien , L. Zhang , ACS Nano 2018, 12, 109. 29216423 10.1021/acsnano.7b07720 PMC5859122

[92] C. Hu , R. Fang , K. Wang , B. Luk , S. Thamphiwatana , D. Dehaini , P. Nguyen , P. Angsantikul , C. Wen , A. Kroll , C. Carpenter , M. Ramesh , V. Qu , S. Patel , J. Zhu , W. Shi , F. Hofman , T. Chen , W. Gao , K. Zhang , S. Chien , L. Zhang , Nature 2015, 526, 118. 26374997 10.1038/nature15373 PMC4871317

[93] Y. Song , Z. Huang , X. Liu , Z. Pang , J. Chen , H. Yang , N. Zhang , Z. Cao , M. Liu , J. Cao , C. Li , X. Yang , H. Gong , J. Qian , J. Ge , Nanomed. Nanotechnol. Biol. Med. 2019, 15, 13.

[94] L. Chen , Z. Zhou , C. Hu , M. F. Maitz , L. Yang , R. Luo , Y. Wang , Research 2022, 2022, 9845459. 35118420 10.34133/2022/9845459 PMC8791388

[95] V. Muzykantov , Expert Opin. Drug Delivery 2010, 7, 403. 10.1517/17425241003610633 PMC2844929 20192900

[96] X. Yang , Y. Yang , F. Gao , J. Wei , C. Qian , M. Sun , Nano Lett. 2019, 19, 4334. 31179709 10.1021/acs.nanolett.9b00934

[97] A. Thielen , S. Zeerleder , D. Wouters , Blood Rev. 2018, 32, 280. 29397262 10.1016/j.blre.2018.01.003

[98] D. Kalyane , N. Raval , R. Maheshwari , V. Tambe , K. Kalia , R. Tekade , Mater. Sci. Eng. C 2019, 98, 1252. 10.1016/j.msec.2019.01.066 30813007

[99] J. Wu , J. Pers. Med. 2021, 11, 771.34442415

[100] T. Yoshikawa , Y. Mori , H. Feng , K. Phan , A. Kishimura , J. Kang , T. Mori , Y. Katayama , Int. J. Pharm. 2019, 565, 481. 31102802 10.1016/j.ijpharm.2019.05.043

[101] H. Kobayashi , P. Choyke , Nanoscale 2016, 8, 12504. 26443992 10.1039/c5nr05552k PMC4824660

[102] A. Nel , E. Ruoslahti , H. Meng , ACS Nano 2017, 11, 9567. 29065443 10.1021/acsnano.7b07214

[103] A. Saisyo , H. Nakamura , J. Fang , K. Tsukigawa , K. Greish , H. Furukawa , H. Maeda , Colloids Surf. B 2016, 138, 128. 10.1016/j.colsurfb.2015.11.032 26674841

[104] U. Prabhakar , H. Maeda , R. Jain , E. Sevick‐Muraca , W. Zamboni , O. Farokhzad , S. Barry , A. Gabizon , P. Grodzinski , D. Blakey , Cancer Res. 2013, 73, 2412. 23423979 10.1158/0008-5472.CAN-12-4561 PMC3916009

[105] K. Sano , T. Nakajima , P. Choyke , H. Kobayashi , ACS Nano 2013, 7, 717. 23214407 10.1021/nn305011p PMC3586604

[106] Y. Kim , M. Lobatto , T. Kawahara , B. Lee Chung , A. Mieszawska , B. Sanchez‐Gaytan , F. Fay , M. Senders , C. Calcagno , J. Becraft , M. Tun Saung , R. Gordon , E. Stroes , M. Ma , O. Farokhzad , Z. Fayad , W. Mulder , R. Langer , Proc. Natl. Acad. Sci. U. S. A. 2014, 111, 1078. 24395808 10.1073/pnas.1322725111 PMC3903216

[107] M. Lobatto , C. Calcagno , A. Millon , M. Senders , F. Fay , P. Robson , S. Ramachandran , T. Binderup , M. Paridaans , S. Sensarn , S. Rogalla , R. Gordon , L. Cardoso , G. Storm , J. Metselaar , C. Contag , E. Stroes , Z. Fayad , W. Mulder , ACS Nano 2015, 9, 1837. 25619964 10.1021/nn506750r PMC4492477

[108] L. Gao , H. Wang , L. Nan , T. Peng , L. Sun , J. Zhou , Y. Xiao , J. Wang , J. Sun , W. Lu , L. Zhang , Z. Yan , L. Yu , Y. Wang , Bioconjugate Chem. 2017, 28, 2591. 10.1021/acs.bioconjchem.7b00428 28872851

[109] M. Gao , C. Liang , X. Song , Q. Chen , Q. Jin , C. Wang , Z. Liu , Adv. Mater. 2017, 29, 1701429. 10.1002/adma.201701429 28722140

[110] Y. Wang , K. Zhang , X. Qin , T. Li , J. Qiu , T. Yin , J. Huang , S. McGinty , G. Pontrelli , J. Ren , Q. Wang , W. Wu , G. Wang , Adv. Sci. 2019, 6, 1900172. 10.1002/advs.201900172 PMC6662054 31380165

[111] A. Saliba , I. Vonkova , A. Gavin , Nat. Rev. Mol. Cell Biol. 2015, 16, 753. 26507169 10.1038/nrm4080

[112] W. Al‐Jamal , K. Kostarelos , Acc. Chem. Res. 2011, 44, 1094. 21812415 10.1021/ar200105p

[113] H. Pick , A. Alves , H. Vogel , Chem. Rev. 2018, 118, 8598. 30153012 10.1021/acs.chemrev.7b00777

[114] K. Ahmed , S. Hussein , A. Ali , S. Korma , Q. Lipeng , C. Jinghua , J. Drug Targeting 2019, 27, 742. 10.1080/1061186X.2018.1527337 30239255

[115] S. Himbert , M. Blacker , A. Kihm , Q. Pauli , A. Khondker , K. Yang , S. Sinjari , M. Johnson , J. Juhasz , C. Wagner , H. Stöver , M. Rheinstädter , Adv. Biosyst. 2020, 4, 1900185. 10.1002/adbi.201900185 32293142

[116] Y. Song , N. Zhang , Q. Li , J. Chen , Q. Wang , H. Yang , H. Tan , J. Gao , Z. Dong , Z. Pang , Z. Huang , J. Qian , J. Ge , Chem. Eng. J. 2021, 408, 127296.

[117] D. Pegtel , S. Gould , Annu. Rev. Biochem. 2019, 88, 487. 31220978 10.1146/annurev-biochem-013118-111902

[118] R. Kalluri , V. LeBleu , Science 2020, 367, aau6977. 10.1126/science.aau6977 PMC7717626 32029601

[119] R. Isaac , F. Reis , W. Ying , J. Olefsky , Cell Metab. 2021, 33, 1744. 34496230 10.1016/j.cmet.2021.08.006 PMC8428804

[120] M. Mathieu , L. Martin‐Jaular , G. Lavieu , C. Théry , Nat. Cell Biol. 2019, 21, 9. 30602770 10.1038/s41556-018-0250-9

[121] Z. Zhang , B. Buller , M. Chopp , Nat. Rev. Neurol. 2019, 15, 193. 30700824 10.1038/s41582-018-0126-4

[122] G. Nam , Y. Choi , G. Kim , S. Kim , S. Kim , I. Kim , Adv. Mater. 2020, 32, 2002440. 10.1002/adma.202002440 33015883

[123] K. Sato , F. Meng , S. Glaser , G. Alpini , J. Hepatol. 2016, 65, 213. 26988731 10.1016/j.jhep.2016.03.004 PMC4912847

[124] L. Barile , T. Moccetti , E. Marbán , G. Vassalli , Eur. Heart J. 2017, 38, 1372. 27443883 10.1093/eurheartj/ehw304

[125] B. Yang , Y. Chen , J. Shi , Adv. Mater. 2019, 31, 1802896.

[126] P. Tran , D. Xiang , T. Tran , W. Yin , Y. Zhang , L. Kong , K. Chen , M. Sun , Y. Li , Y. Hou , Y. Zhu , W. Duan , Adv. Mater. 2020, 32, 1904040. 10.1002/adma.201904040 31531916

[127] O. Betzer , N. Perets , A. Angel , M. Motiei , T. Sadan , G. Yadid , D. Offen , R. Popovtzer , ACS Nano 2017, 11, 10883. 28960957 10.1021/acsnano.7b04495

[128] H. Qi , C. Liu , L. Long , Y. Ren , S. Zhang , X. Chang , X. Qian , H. Jia , J. Zhao , J. Sun , X. Hou , X. Yuan , C. Kang , ACS Nano 2016, 10, 3323. 26938862 10.1021/acsnano.5b06939

[129] S. Hu , X. Wang , Z. Li , D. Zhu , J. Cores , Z. Wang , J. Li , X. Mei , X. Cheng , T. Su , K. Cheng , Nano Today 2021, 39, 101210. 34306170 10.1016/j.nantod.2021.101210 PMC8294084

[130] S. Huang , G. Huang , Future Med. Chem. 2019, 11, 1659. 31469330 10.4155/fmc-2018-0586

[131] L. Li , Z. Bai , P. Levkin , Biomaterials 2013, 34, 8504. 23932249 10.1016/j.biomaterials.2013.07.053

[132] H. Su , Y. Liu , D. Wang , C. Wu , C. Xia , Q. Gong , B. Song , H. Ai , Biomaterials 2013, 34, 1193. 23168385 10.1016/j.biomaterials.2012.10.056

[133] G. Brown , J. Willment , L. Whitehead , Nat. Rev. Immunol. 2018, 18, 374. 29581532 10.1038/s41577-018-0004-8

[134] T. Wight , M. Kinsella , S. Evanko , S. Potter‐Perigo , M. Merrilees , Biochim. Biophys. Acta Gen. Subj. 2014, 1840, 2441. 10.1016/j.bbagen.2013.12.028 PMC4074575 24401530

[135] M. Clément , G. Basatemur , L. Masters , L. Baker , P. Bruneval , T. Iwawaki , M. Kneilling , S. Yamasaki , J. Goodall , Z. Mallat , Circulation 2016, 134, 1039. 27587433 10.1161/CIRCULATIONAHA.116.022668

[136] L. Ma , T. Liu , M. Wallig , I. Dobrucki , L. Dobrucki , E. Nelson , K. Swanson , A. Smith , ACS Nano 2016, 10, 6952. 27281538 10.1021/acsnano.6b02878

[137] D. You , G. Saravanakumar , S. Son , H. Han , R. Heo , K. Kim , I. Kwon , J. Lee , J. Park , Carbohydr. Polym. 2014, 101, 1225. 24299895 10.1016/j.carbpol.2013.10.068

[138] K. Tsuchiya , N. Nitta , A. Sonoda , A. Nitta‐Seko , S. Ohta , M. Takahashi , K. Murata , K. Mukaisho , M. Shiomi , Y. Tabata , S. Nohara , Int. J. Nanomed. 2012, 7, 2271. 10.2147/IJN.S29417 PMC3356181 22619561

[139] Y. Zhao , C. Jiang , J. He , Q. Guo , J. Lu , Y. Yang , W. Zhang , J. Liu , Bioconjugate Chem. 2017, 28, 438. 10.1021/acs.bioconjchem.6b00600 28004910

[140] M. Bidder , J. Shao , N. Charlton‐Kachigian , A. Loewy , C. Semenkovich , D. Towler , J. Biol. Chem. 2002, 277, 44485. 12200434 10.1074/jbc.M206235200

[141] H. Qiao , Y. Wang , R. Zhang , Q. Gao , X. Liang , L. Gao , Z. Jiang , R. Qiao , D. Han , Y. Zhang , Y. Qiu , J. Tian , M. Gao , F. Cao , Biomaterials 2017, 112, 336. 27788352 10.1016/j.biomaterials.2016.10.011

[142] M. Nishida , J. Miyagawa , S. Yamashita , S. Higashiyama , A. Nakata , N. Ouchi , R. Tamura , K. Yamamori , S. Kihara , N. Taniguchi , Y. Matsuzawa , Arterioscler. Thromb. Vasc. Biol. 2000, 20, 1236. 10807738 10.1161/01.atv.20.5.1236

[143] L. Pham , E. Kim , W. Ou , C. Phung , T. Nguyen , T. Pham , K. Poudel , M. Gautam , H. Nguyen , J. Jeong , C. Yong , S. Park , J. Kim , J. Kim , Biomaterials 2021, 269, 120677. 33503557 10.1016/j.biomaterials.2021.120677

[144] Y. Li , J. Che , L. Chang , M. Guo , X. Bao , D. Mu , X. Sun , X. Zhang , W. Lu , J. Xie , Adv. Healthcare Mater. 2022, 11, 2101788. 10.1002/adhm.202101788 34786845

[145] H. Yang , Y. Song , J. Chen , Z. Pang , N. Zhang , J. Cao , Q. Wang , Q. Li , F. Zhang , Y. Dai , C. Li , Z. Huang , J. Qian , J. Ge , Int. J. Nanomed. 2020, 15, 901. 10.2147/IJN.S224024 PMC7020933 32103945

[146] Y. Shang , C. Ma , J. Zhang , Z. Wang , C. Ren , X. Luo , R. Peng , J. Liu , J. Mao , Y. Shi , G. Fan , Theranostics 2020, 10, 10231. 32929345 10.7150/thno.48410 PMC7481406

[147] V. Golubovskaya , Cancers 2017, 9, 150. 29088081 10.3390/cancers9110150 PMC5704168

[148] V. Golubovskaya , R. Berahovich , H. Zhou , S. Xu , H. Harto , L. Li , C. C. Chao , M. M. Mao , L. Wu , Cancers 2017, 9, 139. 29065481 10.3390/cancers9100139 PMC5664078

[149] M. B. Leick , H. Silva , I. Scarfo , R. Larson , B. D. Choi , A. A. Bouffard , K. Gallagher , A. Schmidts , S. R. Bailey , M. C. Kann , M. Jan , M. Wehrli , K. Grauwet , N. Horick , M. J. Frigault , M. V. Maus , Cancer Cell 2022, 40, 494. 35452603 10.1016/j.ccell.2022.04.001 PMC9107929

